# Microbial processes in geological carbon sequestration: Mechanisms, methods, and engineering implications

**DOI:** 10.1016/j.isci.2026.115792

**Published:** 2026-04-20

**Authors:** Liangchao Huang, Zhengmeng Hou, Jianhua Liu, Tianle Shi, Qichen Wang, Yilin Guo, Lin Wu

**Affiliations:** 1 Institute of Subsurface Energy Systems, Clausthal University of Technology, 38678 Clausthal Zellerfeld, Germany; 2 Research Centre of Energy Storage Technologies, Clausthal University of Technology, 38640 Goslar, Germany; 3 Sino-German Research Institute of Carbon Neutralization and Green Development, Zhengzhou University, Zhengzhou 450001, China; 4 State Key Laboratory of Oil and Gas Reservoir Geology and Exploitation, Southwest Petroleum University, Chengdu 610500, China

**Keywords:** Earth sciences, Biogeochemistry, Biogeoscience, Global carbon cycle, Microbiology, Applied microbiology

## Abstract

Geological carbon sequestration (GCS) is a key option for climate change mitigation, and subsurface microorganisms can alter CO_2_ behavior through biomethanation, bioliquefaction, and biomineralization. This review summarizes the major microbial processes involved in GCS and evaluates their effects on carbon stabilization, resource reutilization, and storage risk. We compare microbial distribution and metabolic functions across representative geological reservoirs and synthesize laboratory, numerical, and field approaches into a multi-scale framework for studying microbially mediated GCS. We also discuss engineering regulation strategies, site selection, monitoring, and risk control, together with current technical challenges and future research priorities. Overall, this review provides an integrated perspective on microbial mechanisms and practical guidance for safer and more effective GCS deployment.

## Graphical abstract

**Figure:**
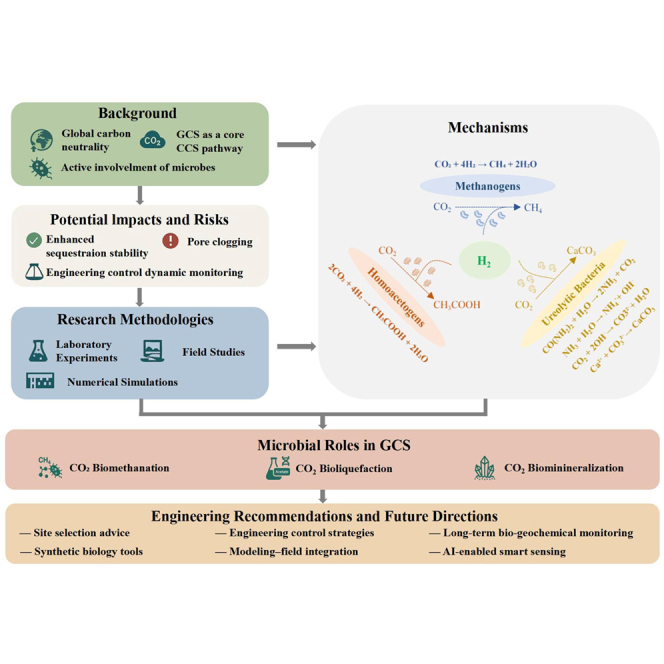

## Introduction

In recent years, the rapid increase in greenhouse gas emissions resulting from large-scale fossil fuel consumption and industrial activities has led to continuously rising atmospheric CO_2_ concentrations. This has exacerbated global warming and intensified climate change challenges.^1^^,^^2^ How to achieve effective carbon emissions reduction and reach net-zero targets, while ensuring economic and societal sustainability, has become a major issue of shared concern among governments and the academic community worldwide.^3^^,^^4^

Carbon capture and storage (CCS) technology is regarded as one of the most important options for addressing climate change. CCS involves separating and capturing CO_2_ generated from emission sources such as coal-fired power plants, steel mills, and cement factories, and subsequently injecting it in a high-pressure or supercritical state into designated storage sites. This process effectively isolates CO_2_ from the atmosphere.^5^^,^^6^ As the core pathway for CCS implementation, GCS primarily relies on geological formations such as deep saline aquifers, depleted oil and gas reservoirs, and deep coal seams for the long-term storage of CO_2_.^7^ However, certain thermophilic, piezophilic, and halophilic microorganisms are capable of surviving under the extreme conditions present in these geological environments. These microorganisms can utilize CO_2_ as an electron acceptor or carbon source, thereby initiating a variety of biogeochemical processes.^8^^,^^9^ For example, field analyses by Tyne et al. demonstrated that indigenous subsurface microorganisms (such as methanogens) can convert 13%–19% of injected CO_2_ into methane, a process known as bio-methanation CCS.^10^ This not only enhances the stability of CO_2_ sequestration but also provides the reservoir with potential value for energy recovery.^11^^,^^12^

Beyond methanogens, other microorganisms, including acetogenic bacteria and ureolytic bacteria, exhibit active metabolic behaviors during CO_2_ sequestration and can profoundly impact the reservoir’s carbon cycle and geochemical environment through diverse metabolic pathways. For instance, acetogens can utilize the Wood-Ljungdahl pathway to reduce CO_2_ with hydrogen to generate acetate, thereby supplying substrates for subsequent methanogenesis or iron(III) reduction.^13^^,^^14^ Ureolytic bacteria can hydrolyze urea, releasing ammonia (NH_3_) and generating carbonate ions (CO_3_^2−^), which significantly increases pore water alkalinity and induces carbonate mineral precipitation. This process can enhance mineral sequestration but may also pose risks of wellbore and reservoir pore clogging and scaling.^15^^,^^16^ Therefore, while microorganisms play indispensable roles in the biogeochemical processes of CO_2_ sequestration, their activity can also have negative impacts. A thorough understanding of microbial community dynamics and metabolic mechanisms is thus essential for ensuring the sustainability and safety of geological carbon sequestration.

To date, numerous review articles have addressed microbial processes in GCS. However, most have only briefly mentioned the basic activities of microorganisms and their potential risks.^17^^,^^18^ In addition, some studies have incorporated microbial processes as secondary components in discussions of numerical simulations, mineral reactions, or field applications.^19^^,^^20^ In recent years, with advances in multi-omics, synthetic biology, and geochemical simulation techniques, a few reviews have begun to focus on the mechanisms of microbial involvement under GCS conditions. For example, Hou et al.^11^ emphasized the use of indigenous methanogens for *in situ* CO_2_-H_2_ biomethanation in depleted oil and gas reservoirs. Zhu et al.,^21^ from the perspective of the deep microbial biosphere, systematically summarized the physiological responses and community succession of microorganisms under high-pressure, high-salinity, and low-pH conditions, as well as their synergistic roles and research gaps in key processes such as mineral precipitation and methane production. Ni et al.^9^ focused on carbonic anhydrase-producing bacteria, elucidating how this enzyme accelerates carbonate precipitation by catalyzing the CO_2_ hydration reaction and providing nucleation sites for carbonate minerals. Nevertheless, existing reviews have yet to systematically categorize the various microbial processes in GCS or provide a comprehensive assessment of their application potential and associated challenges.

**Figure 1:**
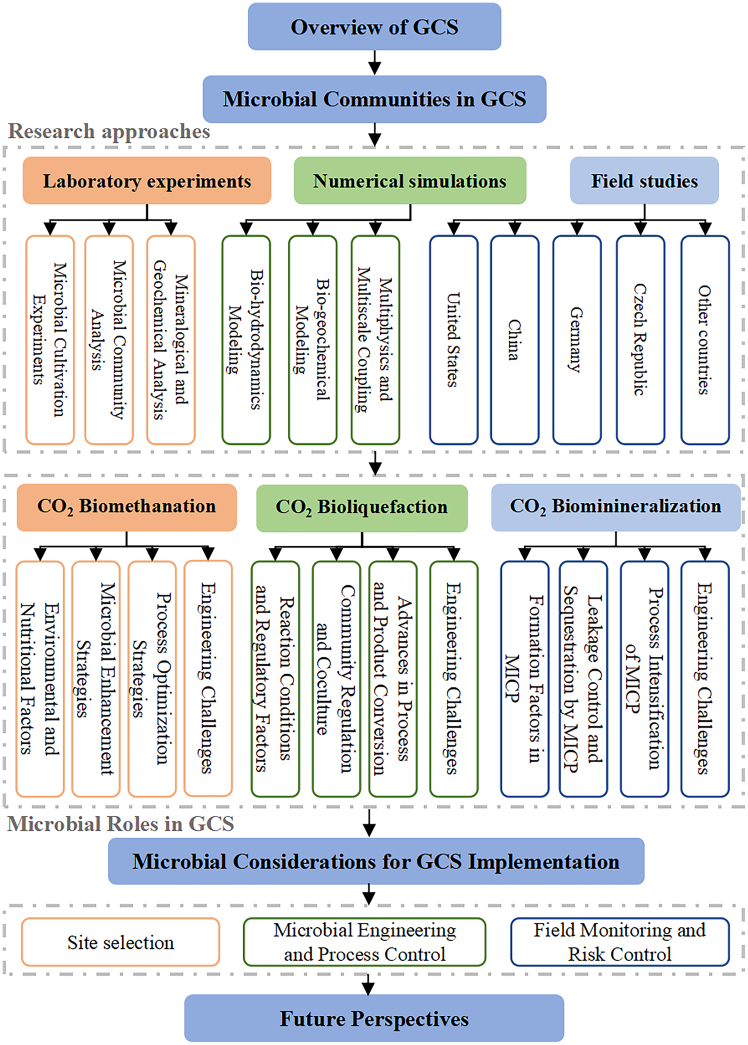
Framework and structure of this review on microbially mediated GCS

Evidently, previous reviews have left significant gaps regarding the reaction pathways, functional types, and engineering implications of microbial participation in GCS, resulting in an incomplete understanding of the interactions among microorganisms, geochemistry, and fluid dynamics. This article aims to review the main mechanisms, research methodologies, and engineering prospects of microbial processes in geological carbon sequestration, covering topics from molecular mechanisms to field cases and from experimental simulations to engineering applications (see Figure 1), with the goal of providing theoretical and technical support from a microbiological perspective for safer, more efficient, and controllable carbon sequestration strategies. The main contributions of this article can be summarized as follows.

• This review systematically classifies and summarizes typical microbial processes in GCS, including mechanisms such as biomethanation, bioliquefaction, and biomineralization.
• It provides a comprehensive overview of the geochemical context, environmental constraints, and technical implementation conditions of microbially mediated carbon sequestration, offering theoretical support for understanding both the facilitative and disruptive effects of microorganisms.
• The proposed research directions and engineering recommendations provide practical guidance for the future application of microbially assisted GCS in diverse geological settings.

The remainder of this review is organized as follows: section 2 introduces the fundamental principles and reservoir types of geological carbon sequestration; section 3 summarizes the characteristics of microbial communities in GCS; section 4 systematically analyzes the research methodologies for investigating various microbial processes in GCS, including laboratory analyses, numerical simulations, and field studies; section 5 discusses the mechanisms of microbial action in GCS, including biomethanation, bioliquefaction, and biomineralization; section 6 offers detailed recommendations regarding site selection, engineering regulation, field monitoring, and risk management for microbially mediated GCS; and section 7 concludes the article and outlines future research trends.

## Overview of GCS

### Fundamental principles of GCS

**Figure 2:**
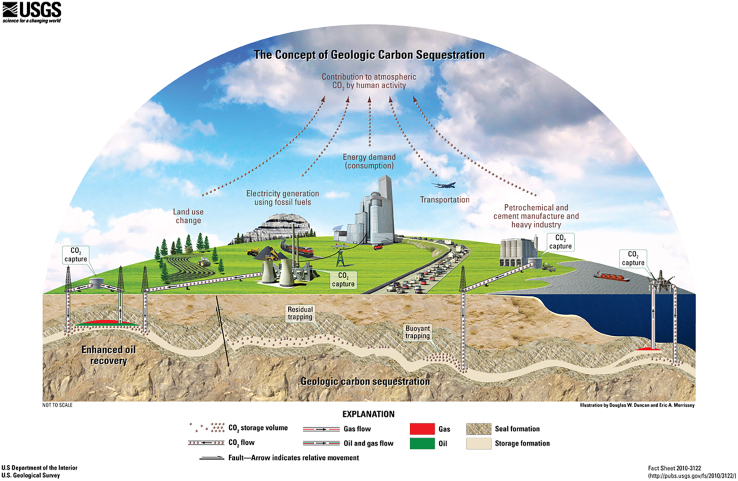
Schematic diagram of GCS (source: US Geological Survey^28^)

CCS is a key technology for reducing CO_2_ emissions from industrial production and energy utilization, and GCS is one of its most widely studied and implemented pathways due to its large storage potential and relatively mature technology.^5^^,^^22^^,^^23^^,^^24^^,^^25^ In GCS, captured CO_2_ (typically in a supercritical state) is injected into deep underground geological formations (e.g., depleted oil and gas reservoirs, deep saline aquifers, and coal seams) for long-term isolation from the atmosphere^26^^,^^27^ (Figure 2).

The long-term storage of CO_2_ in GCS relies on coupled physical and chemical mechanisms.^20^^,^^27^ Physical mechanisms mainly include structural/caprock confinement and pore-scale retention within subsurface formations,^29^^,^^30^ while chemical mechanisms include adsorption, dissolution, ionic transformation, and mineralization.^18^^,^^31^^,^^32^ From the perspective of this review, these processes define the key physicochemical context in which microbial survival, metabolic activity, and microbially mediated effects may occur under different reservoir conditions.

### Common types of geological reservoirs

The selection of a suitable reservoir structure is critical for GCS, as different types of geological reservoirs exhibit distinct characteristics in terms of sequestration mechanisms, storage capacity, and technical applicability (see Table 1). Currently, the most common geological storage sites include deep saline aquifers, depleted oil and gas reservoirs, unmineable coal seams, and basalt formations.^7^^,^^27^^,^^33^ In recent years, several unconventional reservoir types have also emerged, such as salt caverns and CO_2_ hydrate-bearing sediments.^34^^,^^35^^,^^36^

**Table 1:** Comparison of different types of GCS reservoirs

Reservoir Type	Sequestration Mechanism	Storage Capacity	Technological Maturity	Main Advantages	Main Challenges or Limitations
Deep Saline Aquifers^37^^,^^38^	Dissolution storage, mineral reactions, structural trapping, capillary trapping	Extremely large (> several thousand Gt)	High	Large capacity, widespread distribution, high potential	High geological uncertainty, complex long-term monitoring, strict cap rock integrity requirements
Depleted Oil and Gas Reservoirs^39^^,^^40^^,^^41^	Structural trapping, capillary storage, dissolution, mineralization	Medium	High	Mature structure, good sealing, can be integrated with EOR/EGR	Limited capacity, restricted siting, need to assess effects of residual oil/gas on CO_2_ behavior
Unmineable Deep Coal Seams^42^^,^^43^^,^^44^	Surface adsorption	Medium to small	Medium	Strong adsorption capacity, can be coupled with ECBM	Low permeability, coal swelling after injection, high injection/production difficulty
Basalt Formations^45^^,^^46^^,^^47^	Mineral carbonation reactions (CO_2_ mineralization)	Medium to large	Medium	Fast mineralization rate, high storage stability, hydrogen production possible	Limited distribution, difficult control of injection rate, risk of reaction-induced clogging, high water consumption
Salt Caverns^34^^,^^48^^,^^49^	Pressurized cavity injection and structural sealing	Medium	High	Excellent sealing, controllable operation, suitable for short- and long-term storage	Limited individual cavern volume, high construction cost, stringent pressure/structural control requirements
CO_2_ Hydrate- Bearing Sediments^35^^,^^50^^,^^51^	Solid-state sequestration via hydrate crystal structure	Extremely large (theoretical)	Low	High-density storage, strong stability, potential dual benefits (CO_2_ - CH_4_ exchange)	Harsh environmental conditions, high operational difficulty, technology still at research stage

#### Deep saline aquifers

Deep saline aquifers are among the most promising carbon storage reservoirs. Their typical reservoir rocks are sandstone or siltstone, with dense shale layers serving as cap rocks.^37^ Injected CO_2_ rises due to buoyancy and is structurally trapped beneath the cap rock, while a portion of the CO_2_ dissolves in the formation water, reducing leakage risks. Representative projects include Sleipner^52^ and Snøhvit^53^ in Norway, and the Decatur project in Illinois, USA.^54^ These reservoirs are widely distributed worldwide, with a theoretical storage capacity reaching thousands of gigatons. Major challenges include the complexity of long-term monitoring, stringent requirements for cap rock integrity, and limited understanding of coupled geochemical processes within the reservoirs.^38^

#### Depleted oil and gas reservoirs

Depleted oil and gas reservoirs possess well-defined geological structures and inherent sealing properties, and their pore spaces, previously occupied by hydrocarbons, are available for effective CO_2_ storage.^39^ Injected CO_2_ can not only achieve geological sequestration but also be used for enhanced oil recovery (EOR) or natural gas displacement, thereby improving overall economic benefits.^40^ The selection of such reservoirs is relatively mature, but storage capacity is constrained by available pore space, original reservoir pressure, and residual hydrocarbon saturation.^41^

#### Unmineable deep coal seams

For coal seams that are not amenable to mining, CO_2_ injection can exploit its competitive adsorption with methane in coal, enabling the fixation of CO_2_ within the coal matrix while simultaneously displacing methane for enhanced coal bed methane (ECBM) recovery.^42^ This approach can be deployed near emission sources such as power plants, achieving both energy recovery and carbon reduction.^43^ However, coal seams often exhibit low permeability, complex non-linear adsorption-desorption behaviors, and challenges associated with coal matrix swelling and fracture changes during injection, posing significant engineering difficulties.^44^

#### Basalt formations

Basalt, a widely distributed volcanic rock, is rich in calcium, magnesium, and iron, which can react with CO_2_ to form stable carbonate minerals such as calcite and magnesite.^45^ Basalt reservoirs have low leakage risk and high long-term stability, and hydrogen gas can be produced during the sequestration process.^55^ The CarbFix project in Iceland has achieved over 90% mineralization of CO_2_ in basalt within a few years, demonstrating extremely high reaction efficiency and long-term safety.^46^ However, the storage capacity of basalt is limited, the process requires large volumes of water for injection, and the geological structure is complex, necessitating careful control of reaction rates and risks of pore clogging.^47^^,^^56^

#### Salt caverns

Salt caverns are artificial cavities created by solution mining in underground salt rock formations, characterized by extremely low permeability and excellent sealing properties. They have been widely used for the storage of natural gas and hydrogen.^57^^,^^58^ Due to their controllable structure and high integrity, the related construction and operational technologies are relatively mature.^59^ In recent years, salt caverns have also been considered as multifunctional reservoirs for both short- and long-term GCS.^34^ Simulation and engineering studies indicate that, at appropriate burial depths (e.g., 800–1,200 m) and under controlled operational pressures, salt caverns can achieve stable sequestration over hundreds to thousands of years,^48^ meeting the safety and capacity requirements of CCS systems. However, there are limitations, such as restricted individual cavern volumes, high construction costs, and stringent requirements for pressure and structural safety, making salt caverns more suitable for sequestration in regions with favorable resources and concentrated carbon sources.^49^

#### CO_2_ hydrate-bearing sediments

In low-temperature and high-pressure environments, such as deep-sea sediments or permafrost, CO_2_ can combine with water to form stable hydrate crystal structures, enabling solid-state sequestration.^35^ This method theoretically offers extremely high storage density and long-term stability, and can provide dual benefits by enabling the replacement of methane in natural gas hydrates with CO_2_ (CO_2_–CH_4_ exchange).^50^ However, its application is restricted by the need for specific environmental conditions, challenges in controlling temperature and pressure, and complex injection engineering, so it remains in the early stages of research and experimentation.^51^

In addition to geological and engineering differences, reservoir types also differ in microbial adaptability and dominant metabolic potentials, which may influence the likelihood and significance of microbially mediated effects during CO_2_ storage. In deep saline aquifers, the combination of elevated pressure, variable salinity, and low nutrient availability generally favors anaerobic and halotolerant/halophilic microorganisms, while microbial activity is often constrained by limited substrates; under suitable conditions, sulfate reduction, methanogenesis, and iron reduction may still occur.^60^ Depleted oil and gas reservoirs commonly host microbial communities adapted to hydrocarbons and reducing environments, including fermentative microorganisms, methanogens, and sulfate-reducing bacteria, and may therefore show relatively higher potential for gas transformation, souring, or corrosion-related processes when electron donors are available.^61^ Unmineable coal seams provide organic-rich matrices and fracture systems that can support methanogenic consortia and syntrophic metabolisms, making microbial methanogenesis and permeability-related effects particularly relevant in ECBM-associated scenarios.^62^ Basalt formations, by contrast, are mineral-reactive systems rich in Fe-, Mg-, and Ca-bearing phases; microbial processes are often constrained by reservoir conditions and nutrient availability, but iron cycling and microbially influenced mineral precipitation/dissolution may affect CO_2_-water-rock interactions.^63^

For salt caverns and CO_2_ hydrate-bearing sediments, the current understanding of reservoir-specific microbial roles in GCS is more limited and strongly condition-dependent. In salt caverns, extreme salinity and engineered operational conditions may restrict microbial diversity and activity, although halophilic microorganisms and biofilm-related effects cannot be excluded in specific cases. In CO_2_ hydrate-bearing sediments (e.g., deep-sea or permafrost settings), low temperature/high pressure conditions and hydrate stability constraints may select for psychrophilic/pressure-adapted microbial communities, and microbial methanogenesis or methane oxidation may be relevant in some environments, particularly in CO_2_–CH_4_ exchange contexts. Overall, these reservoir-specific differences should be considered together with Table 1 when assessing microbial risks and opportunities in GCS, and their actual expression remains site-specific, depending on local temperature, salinity, pressure, pH, and substrate supply.

### Engineering applications of GCS

As early as the 1970s, the United States began injecting CO_2_ during EOR operations, with the primary objective of increasing hydrocarbon recovery rather than reducing emissions—this was the world’s first large-scale application of CO_2_ for enhanced recovery.^64^ However, the modern concept of GCS as a mitigation strategy was first proposed by Marchetti.^65^ The Sleipner project in Norway is recognized as the world’s first commercial GCS project and is considered a milestone in the field.^5^ Launched in 1996 and operated by Equinor (formerly Statoil), the project aimed to inject CO_2_ separated from natural gas processing into the deep saline Utsira Formation beneath the North Sea, thereby avoiding substantial carbon tax payments.^52^

**Figure 3:**
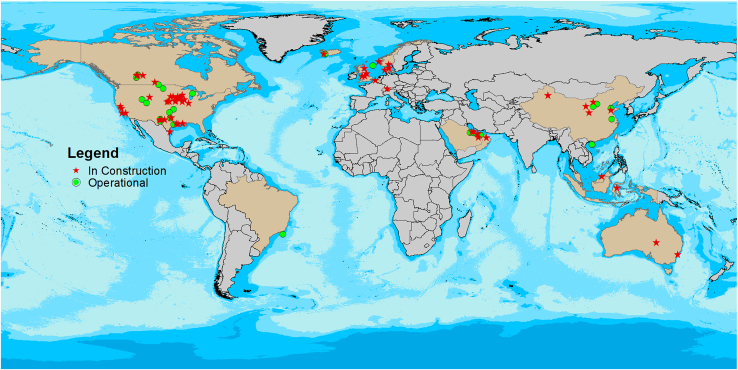
Distribution of GCS projects (data source^66^: Global Status of CCS 2024)

As of July 2024, according to the Global CCS Institute, there are 50 commercial CCS projects in operation worldwide, with another 44 projects under construction and 628 in development—a 60% increase compared to 2023.^66^ Figure 3 illustrates the distribution of operational and under-construction CCS facilities by country. North America and Europe lead globally in policy support and the number of facilities. The Middle East and Africa have set ambitious CO_2_ sequestration targets, with Saudi Arabia planning to capture 44 million tonnes by 2035.^66^ In Asia, particularly China and Southeast Asia, efforts are underway to develop cross-border CO_2_ transport and storage networks. China, for example, has made significant breakthroughs in several demonstration projects, including the world’s largest coal-fired power plant CCS project—the Huaneng 1.5 million tonnes per year facility.^66^

## Microbial communities in GCS

**Figure 4:**
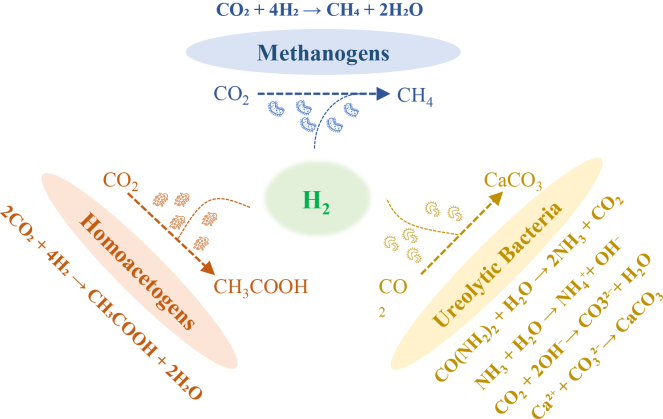
Main processes involving three types of microorganisms in GCS

Compared to surface or shallow environments, deep geological environments are often characterized by extreme physicochemical conditions, such as high pressure, anoxia, elevated temperatures, high salinity, and severe nutrient limitations. As a result, deep microbial communities exhibit unique energy acquisition strategies, metabolic products, and survival tactics. Based on metabolic mechanisms and their influence on geochemical cycles, deep microbial communities can be broadly categorized as follows^67^^,^^68^^,^^69^: methanogens, acetogens, ureolytic bacteria, sulfate-reducing bacteria, nitrate-reducing bacteria, and ferric-reducing bacteria. Among these, methanogens, homoacetogens, and ureolytic bacteria capable of inducing mineralization are most relevant to GCS, with their functional pathways illustrated in Figure 4.

The following sections provide an overview and analysis of the distribution characteristics, metabolic mechanisms, and potential impacts of methanogens, homoacetogens, ureolytic bacteria, and other functional microorganisms in GCS. Table 2 summarizes the optimal and threshold ranges of temperature, pH, and salinity for key microorganisms.

**Table 2:** Environmental Adaptation Parameters for Key Microorganisms in GCS (Summarized and modified based on ref.^70^^,^^71^^,^^72^^,^^73^^,^^74^^,^^75^)

Microbial Type	Environmental Conditions	Temperature Range (°C)		pH Range		Salinity Tolerance (NaCl, g/L)	
		Optimal Range	Threshold Values	Optimal Range	Threshold Values	Optimal Range	Threshold Values
Methanogens	Deep sediments, coal seams, oil reservoirs, other anoxic or anaerobic environments	30–40	Max:122	6.5–7.0	Min:4.0 Max:10.0	<60	Max:200
Homoacetogens	Sediments, anaerobic saline aquifers, anoxic or low-oxygen environments	20–30	Max:72	6.0–7.5	Min:3.6 Max:10.7	<40	Max:300
Ureolytic Bacteria	Soil, shallow strata, nitrogen-rich engineered environments, aerobic or facultative aerobic	20–40	Max:60	7.0–8.0	Min:4.0 Max:12.0	<30	Max:150
SRB	Sediments, oil reservoirs, saline environments, anaerobic conditions	20–30	Max:113	6.0–7.5	Min:0.8 Max:11.5	<100	Max:240
FeRB	Groundwater, reducing sedimentary environments, anaerobic conditions	0–30	Max:90	6.0–7.5	Min:1.6 Max:9.0	<40	Max:200
Carbonic Anhydrase -Producing Microorganisms	Weathered zones, mining soils, carbonate sediment environments, facultative aerobic	20–30	Max:40	9.0–10.0	Min:5.0 Max:13.0	<20	Max:50

The “optimal range” refers to environmental conditions in which microorganisms are most active and metabolically efficient; “threshold values” indicate the tolerance limits under extreme environments.

### Methanogens

Methanogens are members of the domain Archaea and are a group of microorganisms capable of synthesizing methane under strictly anaerobic conditions.^76^ They are widely distributed in deep sediments, coal seams, oil reservoirs, and salt caverns.^77^ Based on their metabolic substrates, methanogens can be classified as hydrogenotrophic, acetoclastic, and methylotrophic types,^78^ among which the hydrogenotrophic type is most representative in GCS environments. The primary reaction catalyzed by hydrogenotrophic methanogens is the Sabatier reaction (CO_2_ + 4H_2_ ⇌ CH_4_ + 2H_2_O, ΔH°_298_ = −164.9 kJ/mol),^79^ which is strongly exothermic. On one hand, increased temperatures enhance the activity of hydrogenotrophic methanogens, forming a positive feedback mechanism; on the other hand, the heat released may cause a significant rise in reservoir temperature, potentially affecting the thermodynamic stability of the formation and the overall safety of the carbon sequestration system.^70^^,^^80^

According to phylogenetic classification, currently known methanogens are divided into four major groups: Methanobacteria, Methanococci, Methanopyri, and Methanomicrobia. Among these, Methanomicrobia is the most diverse group, comprising over 90 identified strains.^70^^,^^77^ These strains display remarkable ecological and metabolic diversity, allowing them to adapt to a wide range of extreme environmental conditions.

In terms of temperature tolerance, methanogens range from psychrophilic to hyperthermophilic. Some strains, such as Methanogenium frigidum, have optimal growth temperatures as low as 15°C, while *Methanopyrus kandleri* can thrive at 98°C, with an upper growth limit reaching up to 110°C.^70^ In geological reservoirs, most methanogens remain active within a temperature range of 30°C–40°C, which is also considered optimal for their growth.^71^ Regarding salinity, the optimal NaCl concentration for most methanogens is below 60 g/L; however, certain species, such as *Methanocalculus halotolerans* and *Methanocalculus natronophilus*, demonstrate exceptional halotolerance and can remain active at salt concentrations up to 200 g/L.^70^^,^^71^ With respect to pH, the vast majority of methanogens prefer neutral to slightly acidic environments (pH 6.5–7.0), although a few strains can tolerate highly acidic (pH 4) or alkaline (pH 9–10) conditions.^70^

### Homoacetogens

Homoacetogens are a group of anaerobic, autotrophic bacteria capable of converting CO_2_ and hydrogen into acetate via the acetogenesis pathway under anoxic conditions.^81^ In subsurface reservoir environments, they are primarily distributed in deep aquifers, oil and gas reservoirs, and deep anaerobic sediments.^12^^,^^82^ Their metabolism mainly relies on the Wood-Ljungdahl pathway (2 CO_2_ + 4 H_2_ → CH_3_COOH +2 H_2_O, ΔH° = −95 kJ/mol),^13^ which is a mildly exothermic reaction. Compared to methanogenesis, this reaction releases less energy but offers greater flexibility in response to environmental changes. In addition, some homoacetogens can synthesize acetate by reducing organic compounds, such as aldehydes and ketones, or by utilizing one-carbon compounds, such as formate and methanol.

Phylogenetically, common homoacetogens include members of the genera *Clostridium*, *Sporomusa*, *Fusobacterium*, and *Acetoanaerobium*.^83^ Among these, *Clostridium* and *Sporomusa* are the most extensively studied, exhibiting strong CO_2_ reduction capacities and broad environmental adaptability.^14^

Homoacetogens demonstrate robust environmental adaptability in their physiological ecology. Their optimal growth temperature typically ranges from 20°C to 30°C, with mesophilic strains (e.g., *Acetobacterium woodii*) and thermophilic strains (e.g., *Moorella thermoacetica*) being the most common. Regarding salinity, most homoacetogens tolerate NaCl concentrations of 0–40 g/L, and some strains remain active in highly saline environments up to 300 g/L. With respect to pH, most strains prefer neutral to slightly alkaline conditions (pH 6.0–7.5), although a few can survive in extremely acidic (as low as pH 3.6) or highly alkaline (up to pH 10.7) environments.^71^^,^^84^

### Ureolytic bacteria

Ureolytic bacteria are microorganisms with urease activity that can catalyze the hydrolysis of urea (CO[NH_2_]_2_), producing ammonia (NH_3_) and CO_2_. The released ammonia reacts with water to generate hydroxide ions (OH^−^) and ammonium ions (NH_4_^+^), thereby increasing the solution pH, promoting carbonate (CO_3_^2−^) formation, and, in the presence of Ca^2+^, leading to the precipitation of calcium carbonate (CaCO_3_),^85^ as illustrated in Figure 4. This capability makes ureolytic bacteria one of the most important functional groups for microbially induced carbonate precipitation (MICP).^15^ In geological settings, ureolytic bacteria can colonize and function in shallow groundwater systems, aquifer margins, carbonate reservoirs, salt cavern walls, and fracture zones, particularly in reservoirs with microfractures or potential CO_2_ leakage risks.^16^^,^^86^

Representative ureolytic bacteria include *Sporosarcina pasteurii*, *Bacillus megaterium*, *Bacillus subtilis*, and *Bacillus sphaericus*.^83^ Among these, *Sporosarcina pasteurii* is the most thoroughly studied and highly efficient ureolytic strain, widely used in MICP research and engineering applications.^16^^,^^87^

The activity of ureolytic bacteria is sensitive to pH, temperature, and salinity. Physiologically, most ureolytic bacteria are facultative anaerobes or aerobes, with optimal growth temperatures between 20°C and 40°C, and no growth observed at 60°C. With respect to salinity, these strains grow best at NaCl concentrations ranging from 2.5 g/L to 30 g/L, although some halotolerant strains (e.g., *B*. *subtilis* BD3) can remain active at concentrations up to 150 g/L. In terms of pH, various strains can grow over a wide range (pH 4.0–12.0), with an optimal range of pH 7.0–8.0, indicating that these bacteria are most competitive under neutral to slightly alkaline conditions.^72^^,^^73^

### Other functional microorganisms

In addition to the three major types of microorganisms discussed above, GCS systems also harbor a variety of functional microorganisms closely associated with CO_2_ migration and mineralization reactions, including the following.

#### Sulfate-reducing bacteria

Sulfate-reducing bacteria reduce sulfate (SO_4_^2−^) to hydrogen sulfide (H_2_S) under anaerobic conditions, using organic matter or hydrogen as electron donors and sulfate as the electron acceptor. A typical reaction is SO_4_^2−^ + 2CH_2_O → H_2_S + 2HCO_3_^−^. This metabolic process produces bicarbonate (HCO_3_^−^) and consumes protons (H^+^), resulting in an increase in local alkalinity and pH, which favors the precipitation of carbonate minerals such as calcium carbonate (CaCO_3_), iron carbonate (FeCO_3_), and magnesium carbonate (MgCO_3_).^88^^,^^89^ However, the metabolic product H_2_S can compete with metal ions and may disrupt the balance between carbonate and sulfide mineral precipitation.^90^ Therefore, SRB can both promote and inhibit mineralization in GCS systems, and their ecological functions are inherently complex.

#### Ferric-reducing bacteria

FeRB use ferric iron (Fe[III]) as an electron acceptor under anaerobic conditions, reducing it to ferrous iron (Fe[II]). The general reaction is CH_2_O + 4Fe(OH)_3_ + 7H^+^ → 4Fe^2+^ + HCO_3_^−^ + 10H_2_O. This process also produces bicarbonate and increases pH, facilitating the precipitation of iron carbonate minerals such as FeCO_3_.^91^^,^^92^ Fe^2+^ can also compete with Ca^2+^ as a cation and, under conditions where SRB are present, can form iron sulfide minerals (e.g., FeS, FeS_2_, etc.), thereby affecting carbonate mineralization efficiency.^93^ Additionally, elevated Fe^2+^ concentrations may alter the local redox potential, further influencing the metabolic activity of other microbial communities.^94^

#### Carbonic anhydrase-producing microorganisms

Carbonic anhydrase is a type of metalloenzyme widely present in various microorganisms, such as *Bacillus*, *Pseudomonas*, and *Xanthobacter*. This enzyme catalyzes the reversible reaction between CO_2_ and water (CO_2_ + H_2_O ⇌ HCO_3_^−^ + H^+^), significantly accelerating the rate of CO_2_ hydration in aqueous environments. As a result, it increases the solubility and reactivity of CO_2_, promoting the precipitation of carbonate minerals.^74^ Compared to urease-based systems, carbonic anhydrase does not produce ammonia, resulting in a milder environmental impact, and is therefore considered a promising low-byproduct technology for facilitating CO_2_ mineralization. However, the activity of carbonic anhydrase depends on metal cofactors such as Zn^2+^ or Co^2+^, and is sensitive to environmental factors including temperature, pH, and salinity. Its stability is particularly limited under high-pressure and high-temperature conditions, and its application currently remains at the experimental validation and engineering optimization stage.^95^^,^^96^

## Research approaches

This chapter summarizes research approaches most relevant to identifying, validating, and monitoring microbial effects in GCS from three perspectives: laboratory experiments, numerical simulations, and field studies. The focus is on GCS-oriented methods (rather than an exhaustive list of geo-microbiological techniques), including deep subsurface sampling and contamination control, microbial and geochemical analyses, biogeofluid/biogeochemical modeling, and multi-scale coupled simulations; where necessary, examples from EOR and related subsurface systems are used with case-by-case caution regarding their transferability to dedicated GCS settings.

### Laboratory experiments

After CO_2_ injection, reservoirs commonly evolve toward extreme conditions (e.g., high pressure, low pH, elevated CO_2_ concentration, and nutrient limitation), which strongly constrain microbial survival and metabolism.^97^^,^^98^ Therefore, laboratory experiments are essential for clarifying microbial adaptive strategies, metabolic pathways, and microbe-rock-fluid interactions under GCS-relevant conditions, and for providing data support for field interpretation and model calibration.

This section focuses on experimental approaches most relevant to GCS-oriented geo-microbiological analysis, including microbial cultivation, community analysis, and mineralogical/geochemical characterization. Table 3 summarizes representative methods, applicable conditions, and major measurement indicators used in GCS and related subsurface studies.

**Table 3:** Comparison of experimental approaches for microorganisms in GCS

Experimental Method Type	Purpose and Key Considerations	Experimental Setup	Environmental Conditions	Microbial Types	Measurement Indicators	Reference
Batch Culture	Changes in microbial community and coal/rock porosity under supercritical CO_2_	High-pressure vessel system (500 mL stainless steel autoclave with temp/pressure control)	35°C; pressure 8, 12, 16 MPa; anaerobic; aqueous	Mixed natural communities (bacteria and archaea, enriched from mine water)	pH, OD600, TDS, salinity, elemental composition of coal sample surfaces, pore structure (liquid nitrogen adsorption)	Li^99^
Flow-Through Culture	Microbe - oil - rock interactions, wettability alteration, reduced IFT, increased oil recovery	Core flow experimental system (carbonate core)	30°C, atmospheric pressure	Bacillus persicus	Changes in permeability, recovery, wettability, IFT, microbial distribution	Haddad^100^
Flow-Through Culture	Microbial migration/growth in pores, bioweathering, pore structure alteration, enhanced permeability and recovery	XIIC-YXLD core flow system (artificial low-permeability core)	30°C; backpressure 8–10 MPa; confining pressure 10–12 MPa	Paenibacillus mucilaginosus	Changes in porosity, permeability, microbial survival, mineral concentrations, recovery	Li^101^
16S rRNA High - Throughput Sequencing	Assessing indigenous denitrifiers’ ability to induce CaCO_3_ precipitation and community dynamics; evaluating GCS safety potential	Illumina MiSeq (PE 2 × 300 bp)	Anaerobic serum vials; 35°C; modified mineralizing medium with Ca^2+^ (∼100 mM) and NO_3_^-^ (∼20 mM)	Indigenous denitrifying bacteria (dominant: Pseudomonas, Dechloromonas)	pH, OD600, Shannon & Simpson diversity indices, community composition	Feng^102^
16S rRNA High - Throughput Sequencing + Metagenomics	Revealing thermophilic spore-former community and function related to deep petroleum seepage; exploring geo-microbiology cycling	Multibeam sonar + sediment corer + anaerobic high-temp bottles + Illumina MiSeq/NovaSeq	High-temp anaerobic seawater medium; 40°C–60°C; N_2_/CO_2_ (90:10) headspace; pasteurization (80°C, 1.5 h)	Thermophilic anaerobic spore-formers (Caminicella, Desulfohalotomaculum, etc.)	Community composition, functional genes (e.g., assA), sporulation genes (spo0A, sspD)	Gittins^103^
Metagenomics	Investigating microbial carbon fixation pathways in deep hydrothermal systems; response to inorganic carbon concentration	750-m hydrothermal well, qSIP culture bottles, ultracentrifuge, Illumina sequencing platform	Neutral to slightly alkaline, 62°C, low O_2_, 1–10 mM NaHCO_3_	Mainly chemoautotrophs (Aquificae, Rokubacteria)	Carbon fixation pathways, MAG abundance, functional gene enrichment (assA, korA)	Coskun^104^
FISH	Monitoring community composition, abundance, and response in saline aquifer pre- and post-CO_2_ injection	647-m deep observation well sampling, FISH slides, fluorescence microscopy, rRNA probe set	Saline aquifer, 35°C, 62 bar, salinity 235 g/L, CO_2_ injection (pH drops to 5.3)	Bacteria, archaea, sulfate-reducing bacteria, methanogens	Cell abundance, bacterial/archaeal ratio, community structure (FISH staining ratios)	Morozova^60^
qPCR	Assessing bacterial/archaeal abundance in high-CO_2_ subsurface; identifying methanogenic response	Subsurface drill samples + anaerobic culture vials	High CO_2_, H_2_ simulated subsurface environment	Archaea (methanogens), bacteria	16S rRNA copy number (bacteria/archaea)	Lipus^105^
XRD +SEM	Mineralogical composition and calcite/aragonite ratio in CaCO_3_ precipitation; crystal morphology	X’Pert^3^ Powder (Netherlands), Zeiss Sigma 500 (Germany)	35°C-55°C; 0.1–7.5 MPa	Sporosarcina pasteurii	Mineral composition (calcite/aragonite), crystal morphology, grain size	Song^106^
XRD, SEM-EDS, XPS, FTIR, ICP-MS, TEM	Mechanism of Cd^2+^ mineralization and precipitate characterization	Bruker D8, Thermo Quattro S, ESCALAB Xi^+^, etc.	30°C, pH 7	B. thuringiensis, C. freundii	Crystal structure, elemental distribution, chemical state	Cai^107^
16S rRNA High - Throughput Sequencing + qPCR + DNA-SIP	Quantifying active methane-oxidizer pmoA gene in oilfield overburden soil; supporting hydrocarbon prospecting	Soil samples + DNA-SIP + qPCR system	Surface soil (oil well vs. dry well areas), 28°C, pH 7-8	Methanotrophs (Methylobacter, Methylosinus)	pmoA gene abundance, community structure	Xu^108^

An important but often underemphasized prerequisite for laboratory analysis of GCS-related samples is deep subsurface sampling quality and contamination control. Drilling fluids, well interventions, and sample retrieval/handling procedures may introduce exogenous microorganisms and labile organic carbon, thereby biasing the interpretation of indigenous community structure and metabolic activity. Therefore, contamination-aware workflows (e.g., tracer-based contamination assessment, sufficient well flushing/conditioning, threshold-based sample acceptance, and careful anaerobic/pH-preserving handling) are important for improving the reliability and comparability of subsequent cultivation, molecular, and geochemical analyses.

#### Microbial cultivation experiments

Microbial cultivation experiments are widely used to evaluate microbial survival, metabolism, and interactions with geological media under simulated GCS conditions, such as high pressure, high CO_2_ concentration, high salinity, and nutrient limitation.^109^^,^^110^ Depending on the experimental system, commonly used approaches include batch cultures and core flow-through experiments.

**Figure 5:**
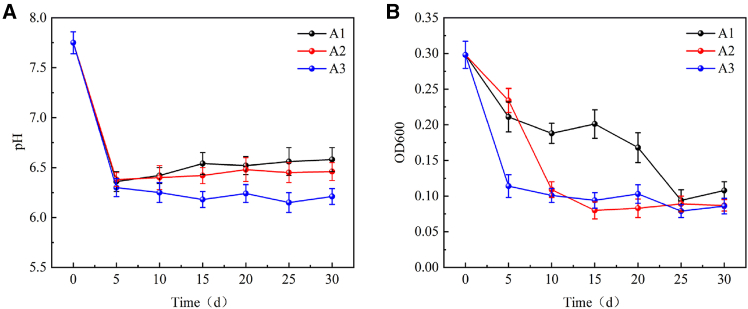
Changes in pH and microbial activity (OD600) during supercritical CO_2_-H_2_O-microorganisms-coal interactions (reprinted with permission from Li et al.^99^ Copyright 2023, Elsevier.)

Batch culture experiments are typically conducted in sealed reactors with controlled temperature, pressure, salinity, and nutrient conditions, and are suitable for screening microbial responses to environmental changes.^99^^,^^111^^,^^112^ For example, Li et al.^99^ established a supercritical CO_2_-water-microorganism-coal system in a high-pressure autoclave and showed that increasing CO_2_ pressure (8–16 MPa) caused a pH decrease, reduced microbial activity (OD600), and significant community restructuring, with pressure-/acid-tolerant genera (e.g., *Paraclostridium* and *Methanoculleus*) becoming dominant (Figure 5).

**Figure 6:**
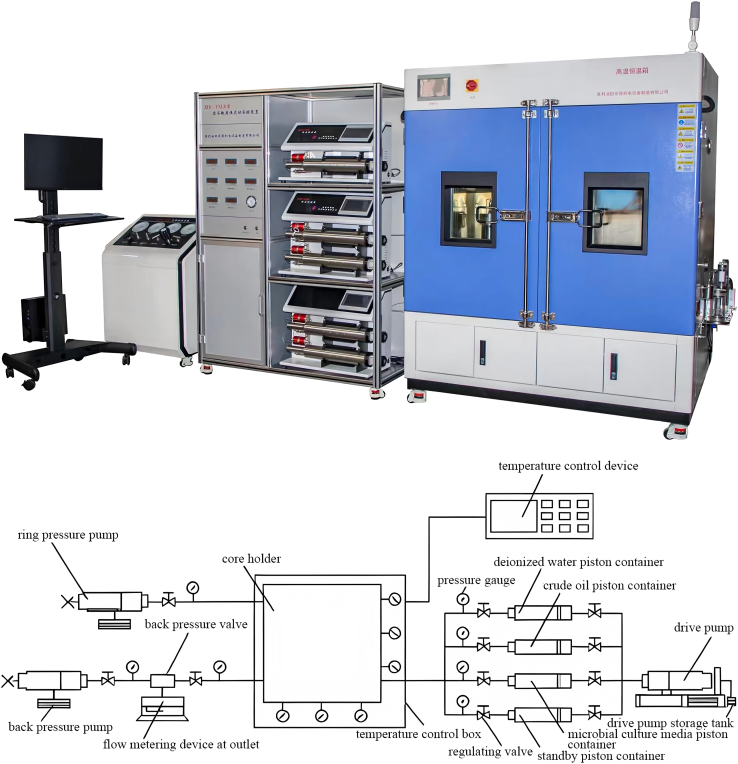
XIIC-YXLD core flow-through experimental apparatus (Shengli Oilfield, Shandong, China. Adapted from Li et al.^101^ Copyright 2025, licensed under CC BY 4.0.)

Compared with batch systems, core flow-through experiments better represent flow and mass-transfer conditions in porous media, and are therefore more suitable for analyzing microbial migration, pore-scale reactions, and porosity/permeability evolution under injection scenarios.^100^^,^^101^^,^^113^ For example, Haddad et al.^100^ reported biosurfactant production and oil recovery enhancement in carbonate cores, while Li et al.^101^ used a core flow system to show that *Paenibacillus mucilaginosus* promoted silicate weathering and increased core porosity/permeability under simulated conditions (Figure 6). Although many such studies are conducted in MEOR-related contexts, they provide useful methodological references for evaluating microbial transport, clogging/weathering effects, and pore-structure evolution in GCS-related porous media experiments.

#### Microbial community analysis

Microbial community analysis techniques are used to characterize community diversity, composition, abundance, and functional potential, and to assess microbial responses to CO_2_ injection and associated geochemical perturbations.^114^^,^^115^ Compared with cultivation-based methods, molecular approaches capture a broader spectrum of uncultivable microorganisms. Commonly used methods include 16S rRNA high-throughput sequencing, metagenomics, FISH, and qPCR.

**Figure 7:**
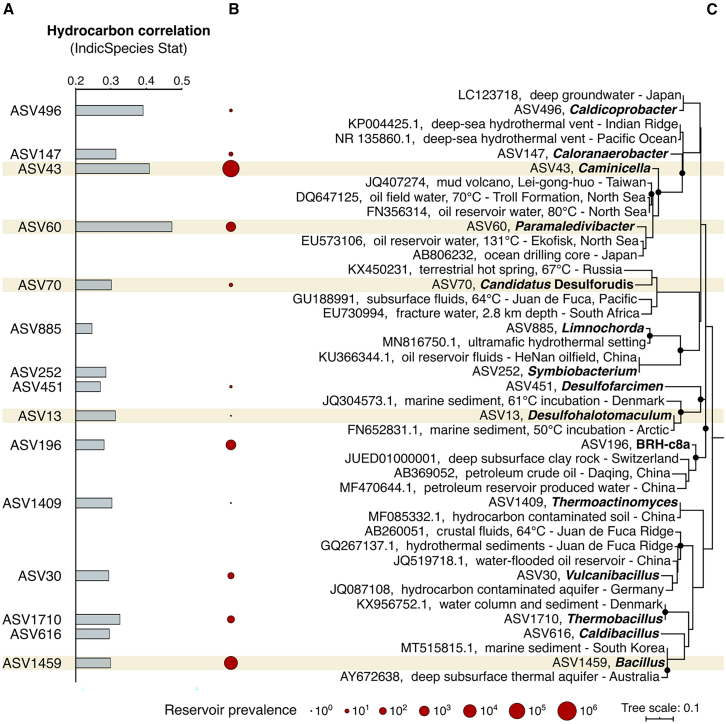
Community features of thermophilic spore-forming microorganisms identified by 16S rRNA gene high-throughput sequencing (adapted with permission from Gittins et al.^103^ Copyright 2022, AAAS.)

16S rRNA high-throughput sequencing is widely used to track bacterial/archaeal community succession before and after CO_2_ injection.^102^^,^^103^^,^^105^ For example, Feng et al.^102^ applied 16S rRNA sequencing to analyze indigenous community evolution during denitrification-induced CaCO_3_ precipitation in a coal seam GCS context, while Gittins et al.^103^ identified thermophilic spore-forming taxa associated with hydrocarbon seep systems (Figure 7), highlighting potential connectivity between deep and surface microbial systems.

Metagenomic sequencing complements 16S rRNA sequencing by revealing functional potential (e.g., carbon fixation, stress resistance, methanogenesis, and sulfur cycling).^103^^,^^104^ For example, Gittins et al.^103^ further identified genes related to anaerobic alkane degradation, fermentation, sulfate reduction, and endospore formation, and Coskun et al.^104^ used quantitative stable isotope probing combined with metagenomic reconstruction to identify deep groundwater microorganisms encoding rTCA and CBB carbon fixation pathways.

FISH provides spatially resolved localization and quantification of target microbial groups in cores and produced waters.^60^^,^^116^ At the Ketzin site, FISH was used to track functional-group responses before and after CO_2_ injection, showing distinct responses of methanogens and sulfate-reducing bacteria to low-pH perturbation.^60^ In current practice, FISH is often combined with sequencing-based methods to verify the *in situ* occurrence and distribution of key taxa identified from molecular data.^116^^,^^117^

**Figure 8:**
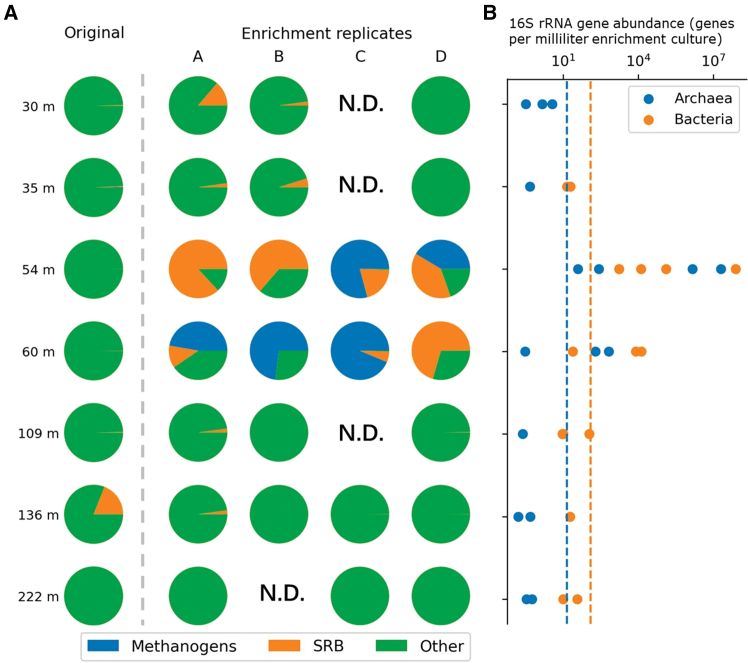
Community structure and gene abundance of methanogenic archaea and sulfate-reducing bacteria at different depths (reprinted from Jia et al.^105^ Frontiers in Microbiology, 2023, licensed under CC BY.)

qPCR is commonly used for sensitive quantification of total microbial abundance (e.g., bacterial/archaeal 16S rRNA genes) and functional genes, and is often applied together with sequencing methods.^105^^,^^108^^,^^118^ For instance, Jia et al.^105^ quantified bacterial and archaeal 16S rRNA genes under high-CO2 subsurface conditions and identified depth-dependent enrichment patterns of methanogenic archaea (Figure 8). In GCS-related studies, the combination of sequencing (community structure) and qPCR (absolute abundance) is particularly useful for evaluating microbial response intensity.

#### Mineralogical and geochemical analysis

Microorganisms in GCS systems can directly or indirectly influence CO_2_-water-rock reactions, causing mineral dissolution/precipitation and changes in pore structure, which in turn affect porosity, permeability, and storage performance.^119^ Accordingly, mineralogical and geochemical analyses are essential for evaluating microbially mediated effects on reservoir evolution.

**Figure 9:**
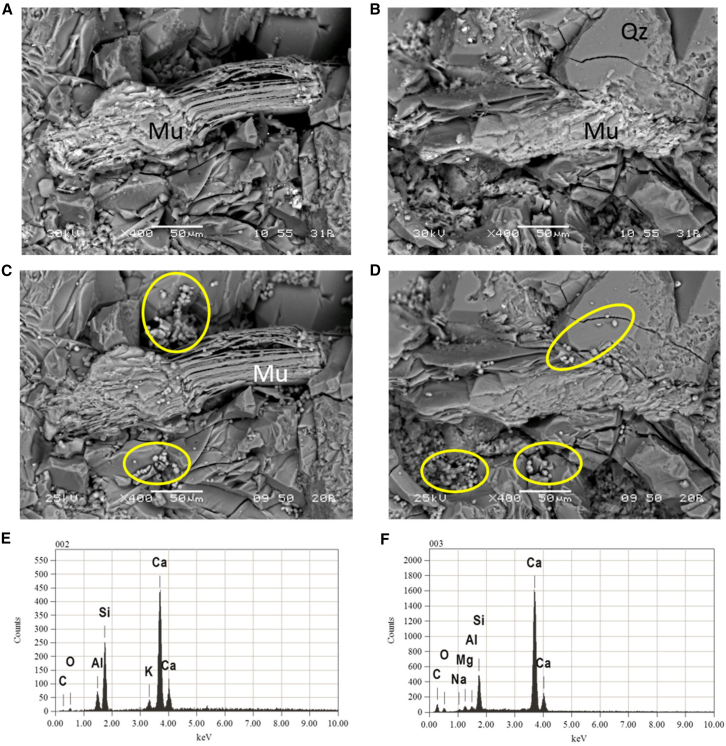
SEM images and EDS spectra of core surfaces before and after reaction (adapted from Pearce et al.^124^ Energies, 2021, licensed under CC BY 4.0.)

Commonly used and complementary techniques include XRD (mineral phase identification), SEM-EDS (microstructure and local elemental composition), and ICP-OES/MS (solution chemistry and elemental migration), often supplemented by XPS, FTIR, SR-μCT, and IRMS for surface chemistry, pore-structure evolution, and isotope tracing. XRD is used to detect mineral phase transformation and newly formed carbonate precipitates,^107^^,^^120^ SEM-EDS provides interfacial-scale evidence of microbial colonization, biofilm formation, and mineral precipitation morphology,^106^^,^^121^ and ICP-based analyses quantify changes in dissolved ions (e.g., Ca, Mg, Na, etc.) and related geochemical effects of microbial processes.^122^^,^^123^ For example, SEM-EDS can directly reveal reaction-induced mineral precipitation and compositional changes on core surfaces; a representative case is shown in Figure 9.^124^

Because no single technique can fully resolve microbially driven geochemical processes, GCS-related laboratory studies increasingly rely on integrated characterization along a “solid phase-interface-fluid-molecular/isotope” chain.^107^^,^^122^^,^^125^ For example, Cai et al.^107^ combined SEM-EDS, TEM, XPS, XRD, FTIR, and ICP-MS to track biomineralization products and metal removal, while Del Buey et al.^125^ integrated XRD, SEM-EDS, and IRMS to jointly constrain mineral precipitation and carbon isotope evolution.

**Figure 10:**
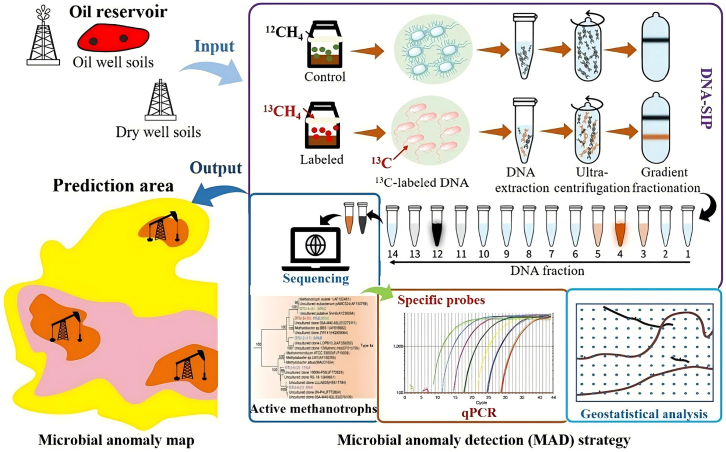
Schematic of the microbial anomaly detection strategy (reprinted from Xu et al.^108^ Copyright 2024, Elsevier.)

Similarly, methods from microbial cultivation, community analysis, and mineralogical/geochemical characterization are often combined to improve interpretation of microbial activity and spatial distribution.^102^^,^^108^^,^^126^ For example, Xu et al.^108^ proposed an integrated microbial anomaly detection strategy (Figure 10) combining qPCR, high-throughput sequencing, DNA-SIP, and geostatistical analysis to identify active methanotrophs in oilfield overburden soils. In recent years, microfluidic techniques have also been introduced into carbon sequestration-related laboratory analyses, enabling high-resolution, real-time observation of CO_2_ transport, multiphase flow, and mineralization reactions at the microscale, and providing new opportunities to investigate fine-scale microbial regulation of CO_2_ storage behavior.^127^

### Numerical simulations

Microbial processes in GCS are complex and strongly coupled with subsurface flow, geochemical reactions, and reservoir evolution. In addition to laboratory experiments, numerical simulations are essential for mechanistic interpretation and long-term prediction.^128^ These approaches enable quantitative description of microbially influenced fluid flow and geochemical dynamics, support extrapolation from laboratory observations to field-scale behavior, and provide a basis for multi-physics and multi-scale coupling. Although developed in an MEOR context rather than specifically for GCS, reservoir-scale microbial modeling approaches implemented in commercial simulators (e.g., using Monod-type kinetics with laboratory-based calibration of microbial growth and metabolite effects) provide a useful methodological reference for coupling microbial processes with subsurface flow and transport simulations, and they also illustrate current challenges in parameterization and model validation.^129^ Table 4 summarizes the main modeling levels, research focuses, representative variables/equations, and typical feedback mechanisms for numerical simulations addressing microbial participation in GCS.

**Table 4:** Key technical levels and features of numerical models

Modeling Level	Main Research Focus	Representative Variables/Equations	Typical Feedback Mechanisms
Biogeofluid Dynamics	How do microorganisms interact with fluids?	*S*_*a*_, *p*, *k*(*φ*), *X*, Monod+Darcy	Flow clogging, gas production/consumption, changes in phase saturation
Biogeochemical Processes	How do microorganisms interact with water-rock chemistry?	*C*_*i*_, Ω_*r*_, *R*_*bio*_, *R*_*c*h*em*_, Monod+Lasaga	pH, Eh, saturation index, mineral dissolution/precipitation
Multi-physics and Multi-scale Coupling	How can flow, chemistry, biology, and thermal-mechanical processes be coupled and scaled up from pore to reservoir?	The above equations plus stress-strain and energy equations; scale mapping	Pressure, thermal expansion/contraction, chemical swelling, hydro-mechanical coupling, parameter upscaling from pore, core, to field

The equations introduced in the following subsections are presented as representative coupling formulations at the framework level, rather than a unique or simulator-specific implementation set; actual variable definitions, constitutive relationships, and thermodynamic treatments may differ across platforms and application scenarios.

To support method selection for different GCS-related research questions, Table 5 provides a comparative overview of commonly used numerical modeling tools for simulating microbially influenced subsurface processes. The comparison focuses on applicability to coupled microbe-fluid-geochemistry-mechanics problems, as well as modeling scope, numerical implementation, user interactivity, and open-source availability, rather than serving as an exhaustive inventory of all reservoir or geo-microbiological simulation platforms.

**Table 5:** Representative numerical modeling tools and key features for microbially influenced subsurface simulations

Software Tool	Biogeofluid Dynamics	Biogeochemistry	Multi-field/Scale Coupling	Model Scale	Advantages	Limitations	Numerical Method	User Interface	Open Source	Reference
PHREEQC	✗	✓	✗	Pore to core scale (1D)	Powerful chemical equilibrium/reaction network modeling; widely used in geochemical and microbial processes	Limited to equilibrium/simple kinetic reactions; only supports 1D flow coupling	Mixed cell method	GUI	Yes	Parkhurst; Mitchell^130^^,^^131^
DuMuX	✓	✓	✓	Pore/core/reservoir scale (3D)	Highly open source; supports flow-reaction–microbial coupling; strong multi-scale extensibility	High computational cost; steep learning curve	Finite volume method (FVM), implicit	No GUI	Yes	Mehmani; Tatomir^132^^,^^133^
TOUGHREACT	✓	✓	✓	Core/reservoir scale (3D)	Mature reactive transport; widely used for multi-field GCS studies	Limited user-defined bioreaction models; advanced features require coding	FVM, implicit/explicit	Limited GUI (version-dependent)	No (restricted)	Vilcáez; Shen^134^^,^^135^
COMSOL Multiphysics	✓	✓	✓	Pore/reservoir scale (3D)	Powerful multiphysics coupling; user-friendly GUI; flexible parameterization	Requires strong numerical modeling skills; high computational resource demand	Finite element method (FEM), implicit	GUI	No (commercial)	Shabani-Afrapoli; Vasile^136^^,^^137^
OpenGeoSys	✓	✓	✓	Pore/core/reservoir scale (3D)	Focused on subsurface problems; open source; robust flow-thermal–chemical-biological coupling	Requires programming and hydrogeology background	FEM, implicit	GUI	Yes	Kolditz; Yin^138^^,^^139^
MRST	✓	✓	Partial	Core/reservoir scale (3D)	Easy to learn in MATLAB; good extensibility; suited for CO_2_ sequestration & microbial modeling in petroleum	Limited multi-field & complex microbe process support; advanced features require custom development	FVM, implicit/explicit	MATLAB GUI	Yes	Safari; Wang^140^^,^^141^
CMG	✓	Partial	Partial	Reservoir scale (3D)	Industrial-grade reservoir simulation; suitable for large-scale EOR/CCS applications	Limited bioprocess/complex geochemical support; low extensibility	Finite difference method (FDM), implicit	GUI	No (commercial)	Jeong; Ansah^142^^,^^143^
ECLIPSE	✓	Partial	Partial	Reservoir scale (3D)	Industry-standard for oil reservoir simulation; reliable and widely used	Very limited microbial/complex geochemical support; low customizability	FDM, implicit	GUI	No (commercial)	Safari; Haq^140^^,^^144^
BioReactPy	✗	✓	Partial	Pore/core scale (1D, 2D)	Open-source Python tool; easy for rapid prototyping; especially for microbe-mineral kinetics coupling	Not widely applied for complex multi-field; lacks flow dynamics module	FDM	No GUI (Python)	Yes	Starnoni; Starnoni^145^^,^^146^

This table provides a representative (non-exhaustive) comparison. Features such as GUI availability and coupling capability may vary by software version, distribution, and user-developed interfaces.

#### Bio-hydrodynamics modeling

In GCS environments, the activities of reservoir microorganisms can induce significant changes in hydrodynamic conditions. Microbes can participate in fluid percolation processes, and through mechanisms such as gas production/consumption, solid precipitation, or biofilm formation, they alter pore structures and fluid properties, thereby affecting the migration and retention of CO_2_ in the subsurface.^147^ Traditional CO_2_ sequestration research has focused primarily on physicochemical processes, often underestimating microbial influences.^84^ However, certain microbial activities (e.g., methanogenic archaea) may play a dominant role in reservoirs and can markedly modify fluid composition and flow dynamics. Thus, it is necessary to incorporate biological processes into reactive flow models to improve predictions of reservoir fluid behavior and CO_2_ fate.

##### Static permeability adjustment models

To reflect microbially induced pore clogging in models, early approaches adopted static parameter adjustment methods. That is, permeability or porosity is empirically or experimentally reduced in advance to mimic the permeability decline caused by microbial growth. For instance, Ivanov et al.^148^ demonstrated in sand column experiments that a numerical model could reproduce dual-stage pore clogging and permeability reduction caused by nitrifying and oligotrophic bacteria by statically lowering the medium’s hydraulic conductivity from 10^−4^ m/s to 10^−6^ m/s. Similarly, Zhong et al.^149^ found that pre-setting medium permeability to about 3% of its initial value could capture the four-stage pore clogging process resulting from sequential aerobic and anaerobic bacterial proliferation. The advantage of this approach lies in its modeling simplicity, as it does not require explicit simulation of microbial processes. Recent microfluidic experiments on microorganism-induced clogging in porous media provide additional mechanistic evidence that permeability reduction is often dynamically linked to biomass growth, biofilm development, and pore-scale flow redistribution, supporting the need for more dynamic formulations of bioclogging in porous-media models.^150^ However, it assumes that permeability reduction is preset and time-independent, failing to capture the spatiotemporal evolution and feedbacks of microbial growth, and thus can only be used for qualitative assessment of worst-case scenarios.

##### Dynamic pore structure feedback models

With growing understanding of microbe-flow interactions, models have evolved to explicitly couple microbial growth kinetics with pore structure evolution, i.e., dynamic feedbacks in biogeofluid dynamics. Here, the motivation is that microbial growth and decay dynamically change porosity, which in turn affects permeability and flow fields. Typically, microbial biomass *X* (or cell concentration *n*) and substrate concentration *S* are added as state variables, and Monod kinetics are introduced to describe microbial proliferation and substrate consumption.^151^ The classical Monod model assumes that the specific microbial growth rate *μ*(*S*) approaches saturation as the limiting substrate *S* increases:

(Equation 1) $$\mu \left(S\right)=\mu_{\max}\frac{S}{{S+K}_{S}}$$

Accordingly, the mass balance equation for biomass (neglecting attachment/detachment) can be written as the following formula:

(Equation 2) $$\frac{\partial X}{\partial t}=Y\mu \left(S\right)X-bX+D\nabla^{2}X$$

where *Y* is the yield coefficient (relating growth to substrate consumption), *b* is the decay rate, and *D* is the diffusion coefficient of biomass. Accumulation of biomass reduces porosity *φ*, which can be related empirically to permeability feedback.

Assuming biomass accumulation occupies pore space, porosity can be updated as a function of biomass volume fraction (or biomass concentration converted to occupied volume through a density/volume conversion factor). In practice, the exact form of this update is model-dependent and depends on how biomass is defined (e.g., mass concentration, cell concentration, or biofilm volume fraction).

Furthermore, an empirical Carman-Kozeny-type relationship can map instantaneous porosity to permeability^152^:

(Equation 3) $$k\left(t\right)=k_{0}{\left(\frac{\varphi \left(t\right)}{\varphi_{0}}\right)}^{n}$$

The key model variables—porosity *φ*(*t*), permeability *k*(*t*), microbial concentration *X*(*t*), and solute concentration *S*(*t*)—thus co-evolve via the above equations. The core assumption is that microbial growth directly reduces the available pore volume and decreases the conductivity of flow pathways, resulting in a dynamically updating clogging feedback over time.

##### Multiphase and multicomponent transport models

GCS systems often involve multiphase flow—such as CO_2_-brine interactions—and reactive transport of dissolved species. Consequently, biogeofluid dynamics models have evolved into multiphase, multicomponent coupled frameworks. The motivation is to accurately simulate the spatial distribution of each fluid phase and chemical component under microbial activity. For example, microbial consumption of dissolved CO_2_ or microbial generation of gaseous products (e.g., CH_4_, N_2_, etc.) can alter both the composition and relative permeability of gas and water phases.

Building upon single-phase models, these frameworks incorporate mass conservation equations for the gas phase (supercritical CO_2_ or generated CH_4_, N_2_, etc.) as well as mass transfer equilibrium between water and gas phases. The typical governing equation for each chemical component *k* can be written as^153^

(Equation 4) $$\frac{\partial }{\partial t}\left[\emptyset \left(\rho_{g}c_{g}^{k}S_{g}+\rho_{\omega }c_{\omega }^{k}S_{\omega }\right)\right]+\nabla \cdot \left(\rho_{\omega }c_{\omega }^{k}v_{\omega }+J_{\omega }^{k}+\rho_{g}c_{g}^{k}v_{g}+J_{g}^{k}\right)=q^{k}$$

where *ρ*_*g*,*w*_ are the densities of the gas and water phases, $c_{g,w}^{k}$ are the mass (or mole) fractions of component *k* in each phase, *S*_*g*,*w*_ are the phase saturations, *v*_*g*,*w*_ are the Darcy velocities, $J_{g,w}^{k}$ are dispersive-diffusive fluxes, and *q*^*k*^ represents microbial reaction sources or sinks (negative for consumption, positive for production).

In addition, the model is closed with momentum conservation (Darcy’s law) and phase equilibrium relationships (e.g., gas-liquid equilibrium). The multiphase, multicomponent model thus introduces fields for phase saturation *S*_*g*_(*x*,*t*), concentrations of multiple species (dissolved substrates and products), and pressure, while also considering feedbacks from microbial activity on medium properties: biomass accumulation reduces porosity and decreases both absolute permeability and effective phase permeabilities, while gas production increases gas-phase saturation, alters relative permeability curves, and may even influence wettability and interfacial tension.^84^ Compared to single-phase models, this framework can simulate the coupled evolution of multiphase flow and component concentrations in scenarios such as “microbial conversion of CO_2_ to methane,” thereby evaluating the integrated impact of microbial activity on multiphase flow in the reservoir.

##### Coupled attachment, gas production, and metabolite transformation models

The latest advances in biogeofluid dynamics modeling have further integrated multiple coupled processes—namely, microbial attachment, gas production, and metabolite transformation—to provide a more comprehensive representation of how microbial activity affects subsurface flow. The modeling motivation is that, in reality, microorganisms exist not only as suspended cells in the fluid phase but also attach to pore surfaces to form biofilms; meanwhile, their metabolic activity may generate gases or solid precipitates. All these processes can induce significant feedbacks on pore structure and fluid composition.

To this end, recent models extend the multiphase, multicomponent framework by adding new state variables and equations. For example, microbial populations may be divided into attached phase *n*_*a*_ (biofilm) and free phase *n*_*d*_ (planktonic cells), with attachment-detachment kinetics described as follows^154^^,^^155^:

(Equation 5) $$\frac{\partial }{\partial t}\left(\varphi S_{\omega }n_{a}\right)=\mu_{gr}S_{\omega }n_{a}-\mu_{dec}S_{\omega }n_{a}+k_{a}S_{\omega }n_{d}-k_{d}S_{\omega }n_{a}$$

(Equation 6) $$\frac{\partial }{\partial t}\left(\varphi S_{\omega }n_{d}\right)+\nabla \cdot \left(n_{d}v_{\omega }-DS_{\omega }{\nabla n}_{d}\right)=\mu_{gr}S_{\omega }n_{d}-\mu_{dec}S_{\omega }n_{d}-k_{a}S_{\omega }n_{d}+k_{d}S_{\omega }n_{a}$$

where *k*_*a*_ and *k*_*d*_ are the attachment and detachment rates, respectively; *μ*_*gr*_ and *μ*_*dec*_ are the Monod-type growth rate and linear decay rate, respectively; *v*_*ω*_ is the water-phase Darcy velocity, and *D* is the dispersion coefficient.

To represent stronger permeability impacts of attached biomass, empirical porosity-reduction relationships (e.g., Langmuir-type forms) are often used, with parameters requiring calibration for the target medium and microbial system^156^^,^^157^:

(Equation 7) $$\varphi =\varphi_{0}\frac{\varphi_{0}-\varphi_{\min}}{1+{\left(n_{a}/B\right)}^{m}}$$

where *φ* is the current (dimensionless) porosity, *φ*_0_ is the initial porosity (prior to microbial impact), *φ*_*min*_ is the residual porosity after complete clogging, and *n*_*a*_ is the concentration of attached microorganisms.

Beyond attachment, models also couple microbial gas production and product precipitation as multiphase reactive mechanisms. For example, in subsurface biomethanation scenarios, the methane component is added and microbial kinetics for the consumption of H_2_ and CO_2_ and the production of CH_4_ and H_2_O are incorporated, with these reactions represented in the mass balance^158^^,^^159^:

(Equation 8) $${CO}_{2}+4H_{2}\to {CH}_{4}+2H_{2}O$$

The newly formed CH_4_ increases gas saturation, which can create bubbles within pore spaces and alter the pressure field and relative permeability. The gas production rate is typically described using (dual) Monod-type kinetics, and gas-liquid equilibrium is handled via Henry’s law.^160^ In GCS applications, CO_2_ solubility in brine is particularly important for dissolution trapping and reactive transport, and its calculation is strongly affected by temperature, pressure, and salinity.

Similarly, for precipitation of metabolic products—such as MICP—the model includes solid mineral components and incorporates kinetic equations for precipitation/dissolution^161^^,^^162^:

(Equation 9) $$\frac{d\varphi }{dt}=-\frac{R_{prec}}{\rho_{solid}}$$

where *R*_*prec*_ is the precipitation rate per unit volume and *ρ*_*solid*_ is the true density of the precipitate. Both increased attached biomass and solid precipitate formation drive a monotonic decrease in porosity *φ* over time, thus reducing medium permeability and intensifying clogging effects.

#### Bio-geochemical modeling

Early bio-geochemical models generally assumed that all chemical reactions could reach equilibrium, calculating the concentrations of reactants and products at equilibrium by solving the law of mass action and mass balance equations.^163^ In GCS scenarios, the injection of large amounts of CO_2_ into saline aquifers or depleted oil and gas reservoirs triggers the coupled evolution of water-rock-gas-microbial interactions. To quantitatively describe this process, researchers initially adopted thermodynamic equilibrium models: all reactions are assumed to reach equilibrium instantaneously, and the law of mass action is used to determine the final distribution in the CO_2_-H_2_O-rock-ion system. This approach can assess whether minerals such as calcite or siderite are supersaturated and thus estimate potential mineral carbon sequestration.^135^^,^^164^ Implementation in programs such as PHREEQC requires only the specification of total element concentrations and equilibrium constants, resulting in high computational efficiency.^165^ The underlying thermodynamic/geochemical database is also a critical model component in such calculations, because database selection affects aqueous speciation, saturation indices, and mineral equilibrium/kinetic calculations; in practice, database choice should be matched to the target mineral system, brine chemistry, and temperature-pressure range. However, because this method ignores reaction rates and microbial metabolism, it cannot describe the propagation of reaction fronts or substrate competition.

With field and high-pressure reactor experiments demonstrating that mineral weathering, microbial metabolism, and solute transport occur on comparable timescales, kinetic rates have been introduced,^134^ establishing a “parallel chemical-biological kinetics” framework. The dissolution-precipitation rates of minerals are typically modeled using the Lasaga formalism^166^^,^^167^:

(Equation 10) $$R_{r}=k_{r}A_{r,s}{\left(1-\Omega_{r}\right)}^{\theta }$$

(Equation 11) $$\Omega_{r}=\frac{Q_{r}}{K_{r,eq}}$$

where *A*_*r*,*s*_ is the specific surface area, *k*_*r*_ and *θ* are calibrated from experimental data.

Microbial metabolism is described by Monod (or dual-Monod) kinetics^168^:

(Equation 12) $$\mu =\mu_{\max}\frac{C_{D}}{K_{D}+C_{D}}\frac{C_{A}}{K_{A}+C_{A}}$$

(Equation 13) $$\frac{dX}{dt}=\mu X-bX$$

Substrate consumption is coupled to biomass growth via the yield coefficient *Y*^169^:

(Equation 14) $$R_{S}=-\frac{1}{Y}dX/dt$$

This framework has been applied to simulate parallel reactions such as sulfate reduction, methanogenesis, and urea hydrolysis-calcium carbonate precipitation, successfully reproducing phenomena such as pH spikes and rapid nucleation in high-pressure reactor experiments.^170^^,^^171^

Subsequent models have emphasized two-way bio-inorganic feedbacks: for example, microbial acid/base production can adjust solution pH and Eh, thereby altering mineral reaction rates, while mineral weathering releases Ca^2+^, Fe^2+^, and other ions that feedback to affect microbial activity.^137^^,^^172^ In the governing equations for each component, this coupling is implemented by solving biological ($R_{i}^{bio}$) and chemical ($R_{i}^{chem}$) source terms in parallel:

(Equation 15) $$\frac{\partial \left(\varphi C_{i}\right)}{\partial t}+\nabla \cdot J_{i}=R_{i}^{bio}+R_{i}^{chem}$$

As field interest shifts from column experiments to the reservoir scale, the models have further evolved into multiphase, multicomponent reactive transport frameworks. Vilcáez^134^ used TOUGHREACT to develop a multiphase, multicomponent bio-geochemical model to simulate the impact of joint CO_2_ and nutrient injection on indigenous microbial activity in deep geological formations. Vasile^137^ introduced a diffusion-limited coefficient into this framework, reproducing the “dissolution-then-precipitation” pattern of CO_2_ mineralization observed in 50-cm sandstone column experiments and extrapolating to predict that, at a 20-m scale over 10 years, carbon sequestration efficiency could increase from 23% to 45%.

The latest trend is to combine this framework with pore-to-field scale bridging and electron equivalence balance. On one hand, micro-CT-lattice Boltzmann modeling can calculate local pH and saturation index at the microscale, which are then upscaled to the representative elementary volume model via scaling factors. On the other hand, explicitly tracking electron flows enables quantification of the competitive efficiency of methanogenesis, sulfate reduction, and other microbial pathways, providing a basis for designing injection strategies.^173^^,^^174^ Through this approach, bio-geochemical models have progressed from static thermodynamic calculations to multi-reaction, multi-field, and multi-scale coupling, offering essential quantitative tools for predicting long-term CO_2_ trapping efficiency, ensuring safety, and assessing microbial risks.

#### Multiphysics and multiscale coupling

In GCS systems, the injection of CO_2_ simultaneously triggers five coupled processes: thermal (T), hydrological (H), biological (B), chemical (C), and mechanical (M) fields. Fluids transport heat as they flow through pores, microbial metabolism alters solution chemistry and generates or consumes gases, mineral dissolution and precipitation modify the pore skeleton, while pressure and thermal expansion feedback to induce rock deformation. If only individual submodules—such as flow-bio or chem-bio—are solved, cross-disciplinary and cross-scale synergistic effects can be missed. Consequently, researchers have proposed fully coupled THB-C/M frameworks and developed parameter upscaling methods bridging the pore to the reservoir scale.

At the Darcy scale, the most common integrated frameworks consist of four sets of governing equations^175^:

(Equation 16) $$\left(\text{Mechanical}\;\text{equilibrium}\right)\nabla \cdot \sigma +\rho g=0,\sigma =C∶\varepsilon -\alpha \left(p-p_{0}\right)I$$

(Equation 17) $$\left(\text{Multiphase}\;\text{flow}\right)\;v_{\alpha }=-\frac{kk_{r\alpha }\left(\varphi \right)}{\mu_{\alpha }}\left(\nabla p_{\alpha }-\rho_{\alpha }g\right)$$

(Equation 18) $$\left(\text{Energy}\right)\;c_{T}\partial_{t}\left(\rho T\right)+\nabla \cdot \left(\rho c_{T}v_{g}T-\lambda \nabla T\right)=Q_{T}$$

(Equation 19) $$\left(\text{Reaction}–\text{transport}\right)\;{\partial_{t}}_{(}\rho_{\alpha }x_{i\alpha })+\nabla \cdot \left(\rho_{\alpha }x_{i\alpha }v_{\alpha }-D_{i\alpha }{\nabla x}_{i\alpha }\right)=R_{i}^{bio}+R_{i}^{chem}$$

Porosity (*φ*) is coupled to strain, mineral transformation, and bioclogging^176^:

(Equation 20) $$\partial_{t}\varphi =\alpha_{v}\partial_{t}\varepsilon_{v}-\frac{1}{\rho_{s}}\left(R_{prec}+R_{bio-solid}\right)$$

where *R*_*prec*_ describes carbonate precipitation kinetics (Lasaga rate law) and *R*_*bio*-*solid*_ denotes the volumetric increase of biofilm or biomass; both jointly alter permeability (*φ*), feeding back to influence multiphase flow.

Pore-scale models resolve the geometry and spatial distribution of processes within the medium. THMCB equations require inputs such as permeability-porosity functions, reaction rate constants, and thermal/mechanical parameters, all of which are strongly dependent on pore geometry under the combined effects of microbes and minerals. Many researchers now use lattice Boltzmann method-reaction coupling to resolve flow-chemical-biological interactions within real pore geometries, capturing local reaction heat, stress concentration, and precipitation zones.^170^^,^^177^ Using volume averaging or multi-grid techniques, effective rates, specific surface areas, and interfacial heat transfer coefficients obtained from microscale simulations can be projected onto the representative elementary volume scale, greatly improving the credibility and comparability of macroscopic simulations.

Notably, microscale simulations have shown that biofilm deposition typically first occurs in low-shear regions, while thermal-chemical-mechanical coupling produces nonlinear amplification effects in these “hotspots.” Neglecting this heterogeneous upscaling in macroscopic models can lead to an underestimation of clogging rates by about 40%.^170^

At scales of tens to hundreds of meters, THB-C/M models have been applied to systematically assess the impact of injection strategies on storage security. Ershadnia et al.^175^ demonstrated via simulation that, in the short term (<5 years), microbially induced mineralization can reduce near-wellbore permeability by an order of magnitude, suppress CO_2_ migration, and enhance capillary-solubility trapping. However, over longer timescales (>30 years), failing to consider fracture-matrix hydro-mechanical coupling and reaction heat can lead to underestimation of caprock strain driven by thermal expansion, causing leakage risk to be underestimated by one to two orders of magnitude. Osselin et al.,^178^ through coupled modeling of serpentine carbonation with biological promotion, found that exothermic reactions could locally increase temperature by 8°C–12°C, enhance dissolution-precipitation rates by 1.3–1.6 times, and form a thermal halo within a 20 m radius. Neglecting thermal coupling led to underestimation of carbon fixation by ∼25%. This cross-field feedback chain (heat → reaction → porosity → flow → heat) is a hallmark of multiphysics-coupled models and constitutes their key value over single-field models.

### Field studies

At present, a number of countries worldwide have conducted GCS field projects of varying scales and reservoir types, accompanied by *in situ* monitoring and analysis of microbial community dynamics and metabolic processes. The United States leads in both project count and per-site injection scale; China follows, with large-scale injections in both coal seams and oil reservoirs. Projects in Germany, the Czech Republic, Japan, and Australia are more limited in scale or number, but each has provided key *in situ* microbial datasets from different geological settings. These field data not only provide critical evidence regarding microbial survival, metabolic activity, and their impact on CO_2_ storage security in real reservoir environments, but also offer important benchmarks for validating and calibrating numerical simulations and theoretical models.

Taken together, these field cases can be compared in terms of reservoir type, monitoring strategy, dominant microbial responses, and engineering implications, which helps clarify both transferable insights and site-specific limitations for microbially informed GCS.

#### United States

The United States has implemented several prominent demonstration projects that include microbial monitoring. Notable examples include the Illinois Basin Decatur Project (IBDP), the Olla Oil Field CO_2_-EOR project in Louisiana, and the Wallula basalt CO_2_ mineralization test in Washington State.

The IBDP, situated in the Lower Mt. Simon Sandstone formation (at ∼2,200 m depth), has injected approximately one million tonnes of CO_2_. Multiple stratified observation wells were drilled near the injection well to monitor aquifer pressure and collect water samples at various depths.^179^ Microbial research at IBDP focuses on the rock-water-microbe interactions that are critical for the success of geological carbon sequestration. Initial investigations assessed the indigenous microbial baseline in deep saline formations; for instance, Dong et al. obtained highly saline formation water from the Mt. Simon reservoir and found extremely low microbial diversity, with >80% of the community belonging to Halanaerobiales (phylum Firmicutes).^180^ Among these, new iron-reducing species such as *Tepidibacillus decaturensis* were isolated, capable of tolerating 50°C, 250 bar, and salinities up to 150 g L^−1^.^181^ Subsequent studies examined how these microbes, following CO_2_ injection, drive mineral dissolution and precipitation via iron and sulfur reduction, thereby altering porosity, permeability, and wellbore/caprock integrity. These works have also developed microbial indicators for long-term monitoring and early warning of reservoir changes.^182^^,^^183^^,^^184^

**Figure 11:**
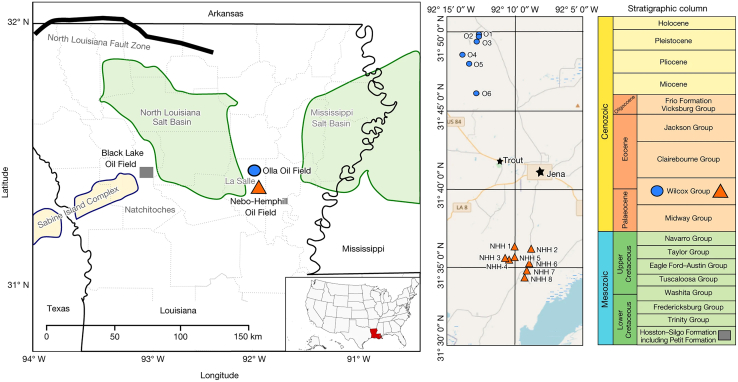
Locations of the Olla and Nebo-Hemphill oil fields as well as the Black Lake Oil Field (reprinted from Tyne et al.,^10^ Nature 2021, licensed under CC BY 4.0.)

In Louisiana, the Olla Oil Field project (Figure 11) utilized downhole sampling and 16S rRNA gene sequencing to show that about 13%–19% of the injected CO_2_ was converted to CH_4_ by indigenous methanogens. This demonstrated the significant impact of CO_2_ injection on the deep reservoir microbiome and highlighted the potential for microbial methanation.^10^

At the Wallula pilot well in Washington, about 1,000 tonnes of supercritical CO_2_ were injected at a depth of ∼800 m. Baseline sampling revealed an initial porewater microbial abundance of 2.1×10^3^ cells mL^−1^; after extended fluid extraction and conditioning, this increased to 6×10^4^ cells mL^−1^, with Proteobacteria dominating (≈89%), alongside functional groups such as the iron-reducing genus *Geoalkalibacter* and the methanotroph *Methylomonas*. More than half of the CO_2_ was mineralized, converting from free/dissolved forms to carbonate minerals. This high mineralization ratio was likely driven by microbially mediated iron reduction and carbonate precipitation, revealing the role of microbes in basalt-hosted mineralization and providing empirical data for the coupled study of geologic sequestration and mineral carbon fixation.^185^^,^^186^

#### China

In recent years, China has actively advanced field studies of CO_2_ sequestration. Representative projects involving microbial processes include the CO_2_-ECBM project in the Qinshui Basin, Shanxi Province, and the large-scale CO_2_-EOR and storage operation in the Jilin Oilfield.

**Figure 12:**
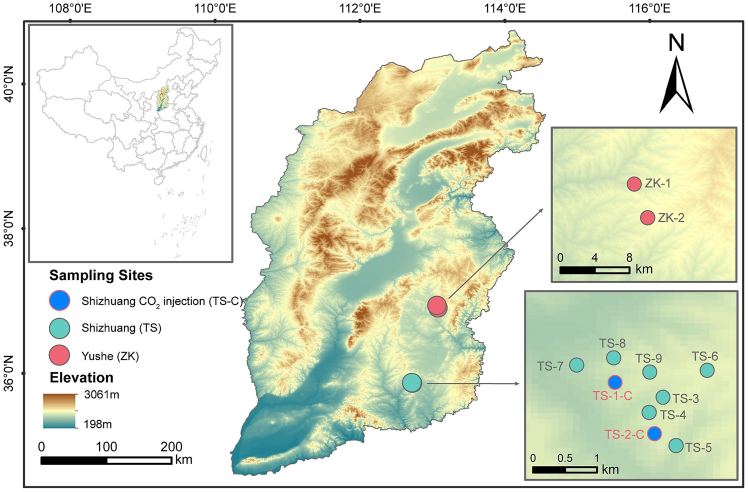
Locations of sampling points for CO_2_-ECBM in the Qinshui Basin (reprinted from Wang et al.^187^ Copyright 2024, Elsevier.)

In the Qinshui Basin (Figure 12), the pilot site targets coal seams at a depth of approximately 560 m, with an injection volume of 0.46 kt CO_2_. Field sampling of groundwater and produced gases revealed a marked increase in methanogen abundance after CO_2_ injection. Isotopic and microbial data indicate that microbially mediated methanogenesis contributed 25%–40% of the produced CH_4_, corresponding to the conversion of approximately 1.1 × 10^4^ mol a^−1^ of injected CO_2_ to methane. These results provide direct field evidence supporting model predictions of microbial CO_2_ conversion.^187^

The Jilin Oilfield project, conducted in continental oil and gas-bearing formations, injects about 800,000 tonnes of CO_2_ annually. Field-collected produced water and aquifer samples were subjected to metagenomic sequencing. Compared with water-flooded zones, microbial α-diversity in CO_2_-flooded zones decreased by 30%, while the abundances of acid- and pressure-tolerant genera—*Methanococcus*, *Halanaerobium*, and *Desulfotomaculum*—increased by 2.1-, 3.4-, and 1.8-fold, respectively. Stable carbon isotope analysis showed that the δ^13^C of dissolved CH_4_ in the EOR wells decreased from −45‰ to −60‰, indicating enhanced hydrogenotrophic methanogenesis. These findings provide direct benchmarks for evaluating competition between microbial methanogenesis and sulfate reduction during large-scale CO_2_-EOR and storage operations.^188^

#### Germany

**Figure 13:**
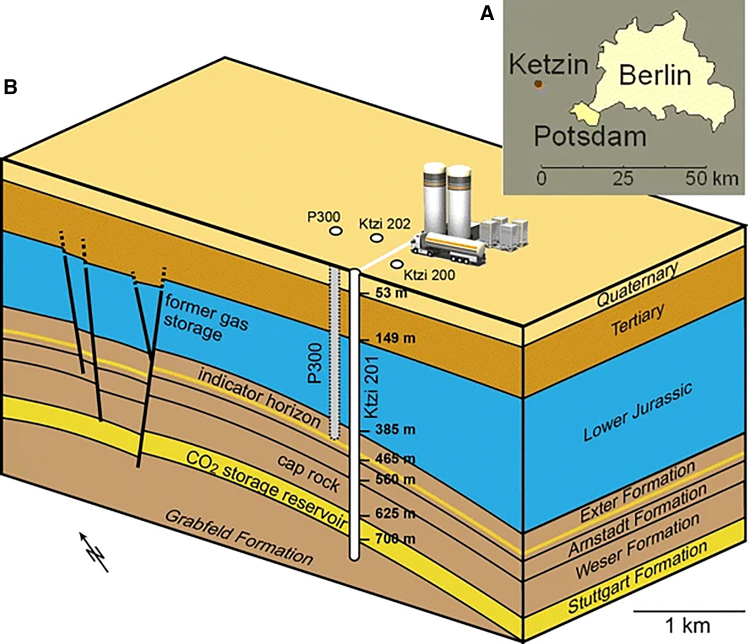
The Ketzin pilot project in Germany, (A) location of the Ketzin site; (B) schematic of the Ketzin anticline structure. (reprinted from Martens et al.^189^ Environ Earth Sci, 2012, distributed under the Creative Commons Attribution License.)

The Ketzin pilot project in Germany is located in Triassic sandstone saline aquifers west of Berlin (see Figure 13). Beginning in 2008, CO_2_ was injected into the Stuttgart Formation at depths of 630–650 m, with a total of 67 kt securely sequestered by 2013. Subsequent monitoring and site management have continued to the present, making Ketzin the earliest and longest-running onshore GCS site in Europe.^189^

At the Ketzin site, researchers implemented rigorous sample contamination control and *in situ* characterization of the “rock-water-microbe” system. Drilling mud was spiked with 1 mg L^−1^ sodium fluorescein and labeled with Rhodamine B to trace fluid movement in real time. Fluorescence microscopy and TOC profiling indicated that mud penetrated a maximum of 20 mm into the core edge; post-drilling hydraulic and N_2_-lift tests extracted 140–190 m^3^ (about 19–21 well volumes), reducing residual mud to 0.04%–0.07%—a quantifiable “clean threshold” for subsequent microbiological sampling.^190^ FISH and 16S rRNA analyses showed that halophilic microbial communities in the reservoir reached abundances of 10^6^ cells mL^−1^, with sulfate-reducing bacteria comprising up to 60% of the community; Proteobacteria and Firmicutes were dominant phyla, and community activity was significantly enhanced by labile organic carbon introduced with the drilling mud.^191^^,^^192^ By contrast, caprock cores from the overlying Exter Formation contained ≤100 cells mL^−1^, mainly Proteobacteria and Actinobacteria, reflecting a deep, carbon-limited native environment.^192^

#### Czech Republic

In two field-scale tests conducted in aquifers and depleted oil and gas reservoirs in the Czech Republic, native methanogenic microbial communities were shown to efficiently catalyze H_2_/CO_2_ conversion on week-to-month timescales. These findings not only explained historical gas storage losses but also provided critical empirical evidence for the coupling of geological CO_2_ sequestration and subsurface biomethanation.

At the Lobodice saline aquifer—the country’s first and only aquifer gas storage facility—city gas (containing 54% H_2_, 12% CO_2_, and 22% CH_4_) was injected in 1989. Within just seven months, a sharp decrease in H_2_ and CO_2_, along with a rise in CH_4_ to ∼40%, was observed.^193^ Concurrently, the δ^13^C-CH_4_ signature shifted from −34.5‰ to −80‰. Coupled with the enrichment of hydrogenotrophic methanogens in formation water (10^3^-10^4^ cells mL^−1^), these results confirmed that the subsurface Sabatier reaction, mediated by hydrogenotrophic methanogens, was the principal mechanism driving H_2_/CO_2_ consumption and CH_4_ generation.^193^^,^^194^

**Figure 14:**
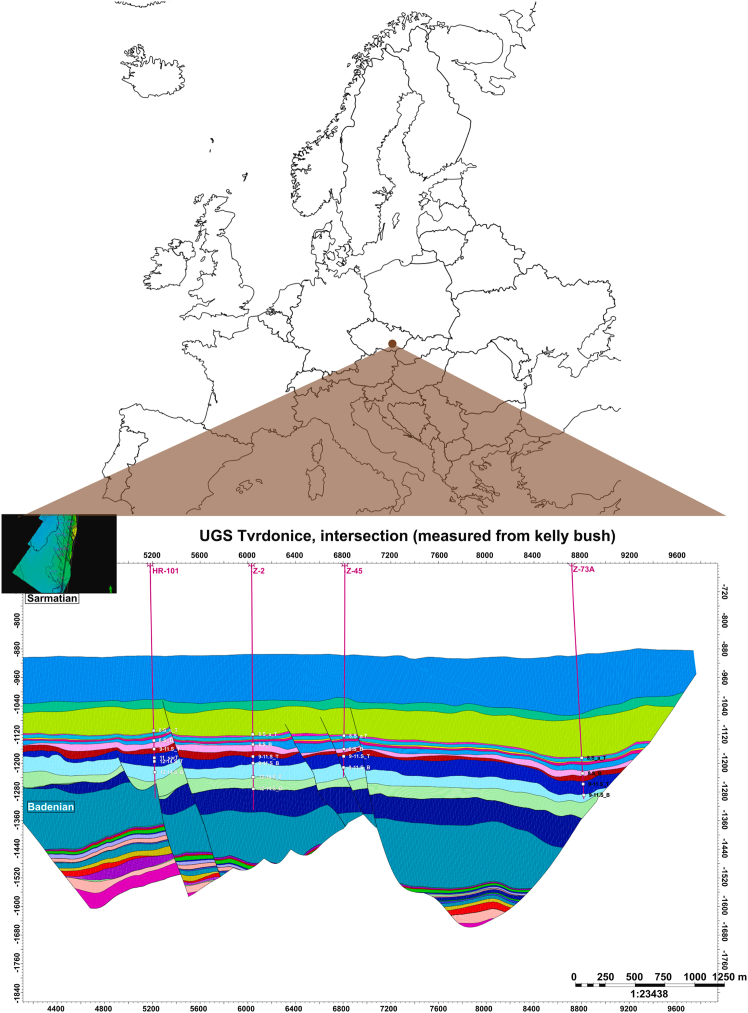
Location of the Tvrdonice natural gas storage site and well Z-73A (reprinted from Vitezová et al.^195^ Copyright 2023, Elsevier.)

The Tvrdonice underground gas storage facility, originally used for natural gas, was converted in 2023 to a mixed-gas (H_2_/CO_2_/N_2_) injection site at a depth of ∼500 m (Figure 14). In the *in situ* “power-to-methane” experiment, well Z-73A was injected with 392 Sm^3^ of synthetic gas (50% H_2_, 12.5% CO_2_, 37.5% N_2_) and 19 m^3^ of formation water. Within 10 days, δ^13^C-CH_4_ rose from −53.6‰ to about −30‰; by day 22, the relative abundance of Methanothermobacter sp. surged to 43% of the microbial community, and both H_2_ and CO_2_ were completely converted to methane within 40 days^195^ Although this project primarily targets underground hydrogen storage, the microbially mediated CO_2_ conversion mechanism closely resembles *in situ* biomethanation in geological carbon sequestration, providing valuable insights and references for future microbial enhancement in GCS.

#### Other countries

Beyond the countries discussed above, many others have launched GCS demonstration projects—including Tomakomai (Japan), Otway (Australia), Sleipner (Norway), Lacq (France), and Acorn (United Kingdom). However, these demonstrations have mainly focused on geological and geochemical monitoring, with systematic microbial monitoring still rare and pending further validation in future projects.^196^^,^^197^^,^^198^ For example, in the Otway Stage 2B injection trial in Australia, deep-well sampling revealed that following CO_2_ injection, the dominant deep subsurface microbial community shifted from Firmicutes to Proteobacteria, with fermentative metabolism remaining active. This indicates that microbes can partially survive and undergo community succession even under supercritical CO_2_ conditions.^199^ Some countries have begun laboratory and modeling studies on microbial responses to high-CO_2_ conditions, preparing for future *in situ* monitoring. For instance, simulated CO_2_ injection into Icelandic basalt formations significantly reduced native microbial diversity while stimulating the proliferation of certain autotrophic groups, highlighting the potential for future field studies.^200^

#### Comparative insights and lessons learned

Across the above field cases, several comparative patterns and lessons can be identified. First, reservoir type strongly controls the dominant microbial response and engineering relevance. In deep saline aquifers (e.g., IBDP and Ketzin), the key issues are commonly related to microbial survival under high salinity/pressure conditions, baseline community characterization, and potential impacts on water-rock reactions, porosity/permeability evolution, and wellbore/caprock integrity. In hydrocarbon-bearing reservoirs and CO_2_-EOR settings (e.g., Olla and Jilin), microbial gas transformation (especially methanogenesis) and competition with sulfate reduction become more prominent due to the presence of reducible substrates and complex redox conditions. In coal-related settings (e.g., Qinshui ECBM), methanogenic enrichment and CO_2_-to-CH_4_ conversion may be more directly observable, while permeability and adsorption-related constraints remain important. In basalt-hosted systems (e.g., Wallula), field observations suggest that microbial processes may interact with mineralization pathways, but the relative contribution of microbes versus abiotic water-rock reactions still requires careful evaluation.

Second, monitoring design strongly influences interpretability. Cases such as Ketzin highlight that contamination control during drilling and sampling is not a minor methodological detail but a prerequisite for reliable microbiological interpretation. By contrast, studies that combine microbial community analysis with isotopes, gas composition, and geochemical indicators (e.g., Olla, Qinshui, Jilin, and leakage-related monitoring studies discussed in Section 6.3) provide more robust evidence for distinguishing microbial transformation from purely physicochemical changes. This suggests that future field studies should prioritize integrated, multi-parameter monitoring and baseline-to-post-injection comparisons rather than relying on single biological indicators.

Third, the current field evidence remains highly heterogeneous in scale, objectives, and monitoring continuity, which limits direct cross-site generalization. Key site conditions controlling microbial relevance (e.g., depth, temperature, pressure, salinity, and substrate availability) are also reported heterogeneously across existing field studies, which further constrains direct comparison and highlights the need for more standardized field reporting. Many projects still emphasize geological and geochemical monitoring, while systematic long-term microbial monitoring remains limited. Therefore, a practical lesson for future work is to strengthen standardized sampling and contamination control, expand long-term multi-parameter observations, and explicitly report reservoir conditions and operational context so that field cases can be compared more rigorously and used more effectively for model validation and risk assessment.

## Microbial roles in GCS

Microbial involvement in GCS is broadly categorized into three principal processes: biomethanation, mediated primarily by methanogenic archaea under anaerobic conditions to convert CO_2_ into methane, thereby enabling the resource utilization of greenhouse gases; bioliquefaction, driven mainly by homoacetogenic bacteria that produce organic acids and other metabolites to locally regulate pH and enhance CO_2_ dissolution in reservoir fluids; and biomineralization, in which specific microorganisms, particularly ureolytic bacteria, induce carbonate precipitation (MICP) to immobilize CO_2_ as stable minerals such as calcite and aragonite. The key characteristics of these processes are summarized in Table 6.

**Table 6:** Main microbial processes and their mechanisms involved in GCS

Microbial Process	Reaction Mechanisms	Representative Microorganisms	Main Products	Functional Roles in GCS
Biomethanation^201^^,^^202^^,^^203^^,^^204^	Methanogenic archaea utilize CO_2_ as an electron acceptor under anaerobic conditions and reduce it to CH_4_.	Methanogenic archaea (e.g., Methanobacterium spp., Methanosarcina spp.)	CH_4_, water	Enables resource recycling of CO_2_; sequestration effectiveness depends on CH_4_ capture systems, posing potential leakage risks.
Bioliquefaction^205^^,^^206^^,^^207^	Homoacetogenic bacteria reduce CO_2_ with H_2_ to soluble organic acids (e.g., acetate), enhancing the biological availability of CO_2_ and indirectly prolonging its retention time in the aqueous phase within reservoirs.	Homoacetogenic bacteria (e.g., Clostridium spp., Sporomusa spp., Acetobacterium spp.)	Dissolved CO_2_, bicarbonate (HCO_3_^−^), organic acids	Increases the solubility of CO_2_ in reservoirs, promoting subsequent mineralization or reducing its mobility through diffusion.
Biomineralization^9^^,^^15^^,^^208^	MICP through ureolytic or sulfate-reducing bacteria modulates alkalinity, releases Ca^2+^/CO_3_^2−^, and forms stable carbonate minerals.	Ureolytic bacteria (e.g., Sporosarcina pasteurii), SRB	CaCO_3_, aragonite, dolomite	Achieves long-term stable solid-phase CO_2_ sequestration, enhances reservoir integrity, and repairs leakage pathways.

### CO_2_ biomethanation

**Figure 15:**
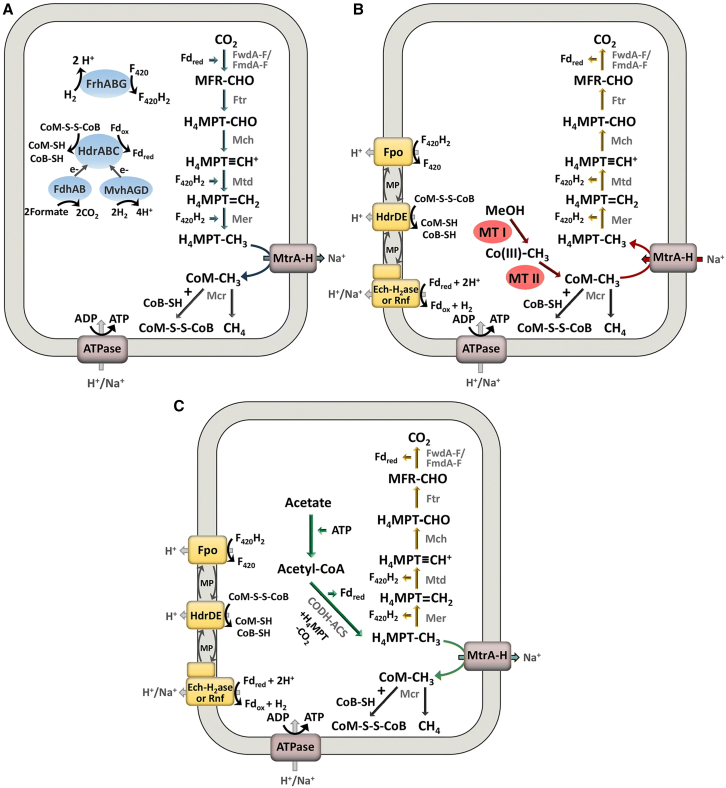
Hydrogenotrophic methanogenesis pathway underlying microbial CO_2_ biomethanation. (reprinted from Kurth et al.^211^ licensed under CC BY 4.0.)

In the context of GCS, CO_2_ biomethanation is primarily mediated by hydrogenotrophic methanogens, which reduce CO_2_ with H_2_ to produce CH4. The underlying metabolic route is shown in Figure 15. Beyond serving as a microbial pathway for CO_2_ conversion and reutilization, this process also offers a potential strategy for storing renewable energy in the form of methane, a storable and transportable energy carrier. During periods of excess renewable energy production, hydrogen can be generated via water electrolysis and subsequently combined with CO_2_ through biocatalysis to produce methane (the so-called “power-to-gas” approach). The resulting methane can then be injected into natural gas grids, thereby enhancing overall energy utilization efficiency.^209^^,^^210^ As an emerging technology, CO_2_ biomethanation is regarded as a promising strategy for harnessing surplus renewable electricity and utilizing waste CO_2_ resources.

#### Environmental and nutritional factors affecting CO_2_ biomethanation efficiency

The efficiency of CO_2_ biomethanation is highly sensitive to environmental conditions. Among these, pH is a critical parameter: most hydrogenotrophic methanogens thrive under neutral to slightly alkaline conditions. Both pH 7.0–7.5 and pH 8.5–9.0 can sustain stable methane production, although the latter often yields a higher maximum methane generation rate, while strongly acidic conditions (pH < 6) significantly prolong the lag phase and suppress microbial activity.^212^

Temperature also plays a vital role; moderate temperatures (ca. 35°C–40 °C) and thermophilic conditions (above 55°C) are both feasible for biomethanation. Raising the temperature generally accelerates microbial metabolism (e.g., studies show methane production at 55°C is approximately twice that at 40°C),^202^ but also decreases CO_2_ solubility by around 30%.^210^ In contrast, the solubility of H_2_ in water is much less sensitive to temperature (declining by only ∼3% from mesophilic to thermophilic conditions), so moderate temperature increases often enhance conversion efficiency within a controllable range.^210^

**Figure 16:**
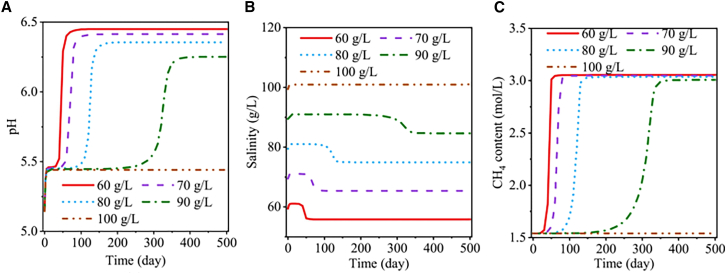
Effect of initial salinity on microbial methanation Effects of initial salinity on (A) pH, (B) salinity, and (C) CH_4_ content (adapted from Wu et al.^158^ licensed under CC BY.)

Salinity is another influential factor. Wu et al.^158^ reported that increasing initial salinity from 60 g/L to 100 g/L significantly inhibited microbial activity, leading to almost no effective methanation within 500 days (see Figure 16).

In addition, hydrogen partial pressure has a pronounced impact on both reaction direction and rate: higher H_2_ partial pressures favor CO_2_ reduction, but excessive H_2_ can cause a rapid pH increase and inhibit the formation of byproducts (e.g., volatile fatty acids), while H_2_ deficiency reduces methane yield or diverts metabolism toward acetate and other byproducts.^212^^,^^213^ Simulation studies have shown that, in carbonate reservoirs, maintaining salinity below 90 g/L and optimizing the injected CO_2_:H_2_ molar ratio to 1:3.78 can enable nearly complete conversion of CO_2_/H_2_ within approximately 110 days, with methane yields of 83%–92% and effective suppression of acetate and H_2_S accumulation.^203^

Finally, nutritional conditions are also essential: sufficient supplies of macronutrients such as nitrogen and phosphorus, as well as trace metals including Ni, Co, Fe, and Mo, are required. These elements are components of key methanogenic enzymes (such as Ni-dependent methyl-coenzyme M reductase, Mcr); deficiencies will limit enzyme activity and methane productivity.^214^

#### Microbial augmentation and community structure regulation strategies

To enhance the stability and rate of CO_2_ biomethanation, researchers have employed various microbial augmentation strategies to construct efficient consortia. One approach is co-cultivation with hydrogen-producing microbes to provide a continuous supply of H_2_ for methanogens. For example, Kato et al.^215^ developed a “co-cultivation” method, growing hydrogenotrophic methanogens alongside hydrogen-producing heterotrophic bacteria. This setup produced a stable, low H_2_ concentration, successfully enriching methanogens adapted to hydrogen-limited environments.

Targeted bioaugmentation has also shown promising results. Feng et al.,^204^ for instance, inoculated a packed-bed reactor with enriched hydrogenotrophic methanogens, leading to the dominance and successful colonization of *Methanobacterium* species, and methane content in the biogas exceeding 96%. In *in situ* co-digestion processes, the addition of *Methanoculleus bourgensis* to mesophilic systems (37°C) increased methane yield by ∼11% and promoted the activity of syntrophic bacteria (such as acidogenic and mutualistic populations), achieving nearly complete conversion of volatile fatty acids.^213^

#### Process optimization strategies

Unlike surface bioreactors, subsurface reservoirs are highly heterogeneous, resulting in strongly uneven distributions of substrates (CO_2_ and H_2_), which significantly affect the spatial profile and overall efficiency of methanogenesis. Therefore, engineering optimization focuses on adjusting injection pressure, well placement, and injection cycles—including pulse or alternating injection schemes—to maximize substrate coverage and utilization in the reservoir. This facilitates effective microbial colonization and dispersal within the porous matrix.^70^^,^^216^

Experimental and numerical studies provide a foundation for optimizing *in situ* methanation. For example, field trials in Austria^82^ and sensitivity analyses^140^^,^^158^ indicate that increasing injection pressure and active biomass levels enhances productivity, whereas high temperature or rapid biomass decay reduce efficiency. Coupled models integrating gas-water two-phase flow and microbial dynamics can guide the optimization of injection rates and cycling parameters to best match specific reservoir conditions, thereby maximizing *in situ* methanation. Furthermore, understanding and managing microbial migration and colonization have become important frontiers for process intensification. Given that natural reservoirs often lack abundant, efficient methanogens, artificial inoculation or *in situ* activation is essential to initiate and sustain biomethanation.^195^^,^^217^

#### Engineering challenges and future perspectives

Despite its promise, large-scale implementation of CO_2_ biomethanation still faces several challenges. First, the reliability and economics of hydrogen supply are major bottlenecks. Most current experiments rely on externally produced hydrogen (via electrolysis), but large-scale production remains costly. For seasonal or grid-scale storage, underground H_2_ injection (e.g., into salt caverns) is proposed, but *in situ* microbes can gradually consume injected H_2_. For instance, studies show that halophilic SRB in salt caverns can oxidize H_2_, generating H_2_S and raising pH, ultimately reducing microbial activity^218^; other research has detected hydrogen oxidation, methanogenesis, and sulfate reduction in brine-saturated caverns.^219^

Second, methane purity and byproduct formation must be addressed. Competing reactions (e.g., acetogenesis from H_2_/CO_2_) can lower methane yields, while the formation of organic acids or sulfides necessitates further purification.^213^^,^^218^ Lastly, scaling up requires robust three-phase (gas-liquid-solid) mass transfer and stable microbial consortia. Further advances in H_2_ mass transfer, byproduct suppression, and integrated system design are needed to meet industrial operational demands.^209^

**Figure 17:**
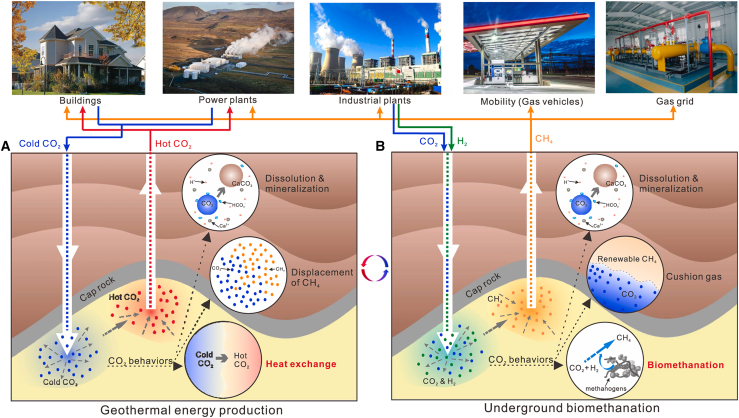
Conceptual illustration of CCCUS (reprinted from Wu et al.^220^ Copyright 2023, Elsevier.)

Future research should target lowering hydrogen production costs, developing strains tolerant of high pressure and contaminants, and better control of byproduct formation, all while incorporating techno-economic analyses. For example, the team led by Michael Zhengmeng Hou proposed an innovative multi-functional negative carbon technology, carbon capture, circular utilization, and sequestration (CCCUS)^11^^,^^220^ (see Figure 17). This technology converts captured CO_2_ and hydrogen-rich industrial byproduct gases into renewable natural gas via underground biomethanation, while also integrating geothermal energy extraction. CCCUS not only enables large-scale CO_2_ recycling, but also combines natural gas storage/switching, geothermal utilization, and geological sequestration functions.^220^^,^^221^ Net present value analysis over a 30-year period further demonstrated the economic viability of this approach.^222^

### CO_2_ bioliquefaction

**Figure 18:**
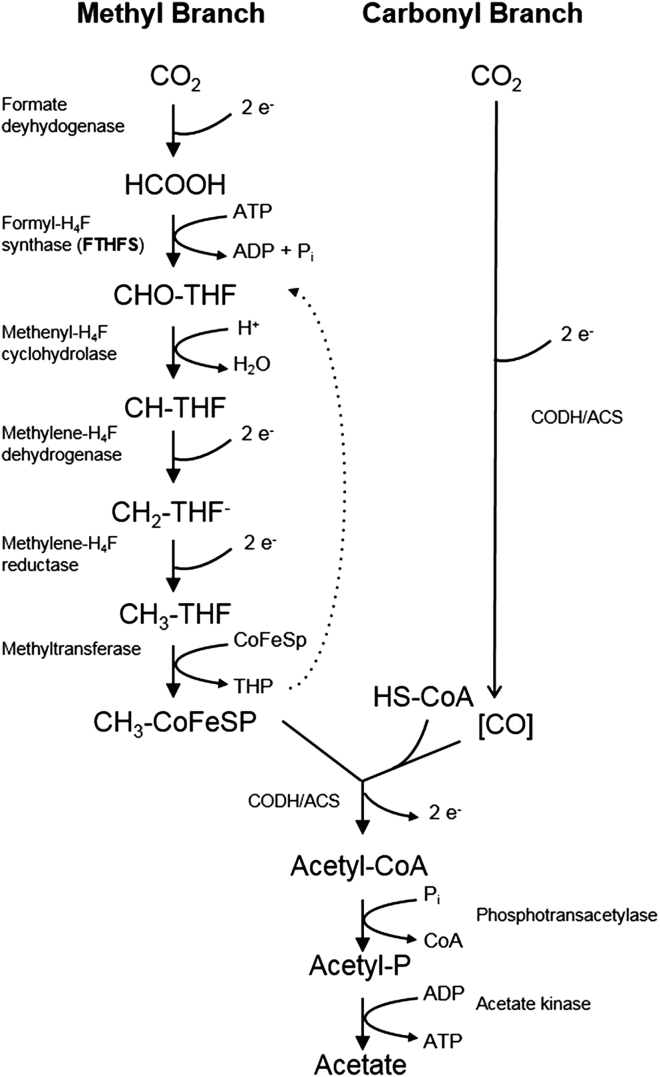
The Wood-Ljungdahl pathway (reprinted under the terms of the license CC BY-NC-ND 4.0 from Westerholm et al.^205^)

CO_2_ bioliquefaction refers to the microbial conversion of CO_2_ into liquid organic products such as acetate and ethanol through metabolic pathways. The underlying mechanisms generally involve the carboxylation and reduction of CO_2_. A classic example is the Wood-Ljungdahl pathway, in which homoacetogenic bacteria (e.g., *Clostridium ljungdahlii*, *Moorella thermoacetica*) efficiently convert CO_2_ to acetate (see Figure 18).^205^^,^^223^ Other fermentative pathways enable the use of CO_2_ as a carboxyl donor to synthesize longer-chain organic acids or alcohols. For instance, propionic acid bacteria can assimilate CO_2_ via the Wood-Werkman cycle to produce propionate, and certain Clostridium species can reduce CO or CO_2_ to ethanol and other liquid products during syngas fermentation.^224^^,^^225^

However, achieving CO_2_ bioliquefaction in deep geological reservoirs—characterized by high pressure, anoxic, hypersaline, and nutrient-poor conditions—remains highly challenging. Supercritical CO_2_, in particular, can acidify microbial cytoplasm and destabilize cell membranes, acting as a potent sterilizing agent. Only microorganisms with specialized resistance mechanisms can survive and remain metabolically active under such extreme conditions.^226^

#### Reaction conditions and regulatory factors

The physicochemical conditions of geological reservoirs directly influence the kinetics of CO_2_ bioliquefaction and microbial activity. Temperature is a critical factor: moderate mesophilic to mildly thermophilic conditions are favorable for the activity of homoacetogenic bacteria, while excessively high temperatures can inhibit their metabolic function. Moreover, temperature modulates the competition between metabolic pathways: at lower temperatures, acetate production is generally favored, whereas methanogenesis becomes increasingly dominant as temperature rises.^14^

pH is another key parameter. Neutral to slightly alkaline conditions typically promote the reduction of CO_2_ to acetate by homoacetogenic bacteria using H_2_ as the electron donor.^206^ However, some studies have shown that under sufficient hydrogen supply, maintaining a mildly acidic pH of around 5.0–5.5 can significantly enhance the conversion of CO_2_ to products such as isobutyrate.^227^

Pressure exerts a dual effect. Elevated pressure increases the solubility of CO_2_ in the aqueous phase, thereby enhancing substrate availability and potentially accelerating the rate of bioliquefaction reactions.^228^ However, when CO_2_ is present as a supercritical fluid, it can exert toxic effects on microbial cells.^226^

Salinity also plays a crucial role. The high salinity of deep saline aquifers imposes osmotic stress on microorganisms. Most known homoacetogens originate from environments with low to moderate salinity, and although some halotolerant species have been reported, extreme salinity typically suppresses microbial activity or leads to cell inactivation.^84^

CO_2_ bioliquefaction generally requires the provision of additional reductants or electron donors to drive the reductive fixation of CO_2_. Hydrogen is the most commonly used and direct electron donor, enabling homoacetogenic bacteria and related species to reduce CO_2_ to organic products under anaerobic conditions.^223^ In addition, the addition of zero-valent iron (Fe^0^) or magnesium (Mg^0^) has been shown to promote acetate formation.^229^^,^^230^ For example, Bayar et al.^229^ demonstrated that the highest acetate concentrations (up to 2 g/L) were achieved with Fe^0^ at 50 or 75 g/L, along with minor ethanol production (125 mg/L). Some microorganisms can also utilize carbon monoxide or formate as electron donors, and in bioelectrosynthesis systems, electroactive microbes can directly acquire electrons from electrodes to reduce CO_2_.^231^

Deep reservoirs are typically highly reducing and anoxic environments, but large-scale CO_2_ injection can disrupt the local geochemical balance. The introduction of acidic fluids can dissolve host rock minerals, releasing previously unavailable electron acceptors such as sulfate, thereby stimulating competing metabolic pathways like sulfate reduction.^110^

#### Microbial community regulation and coculture mechanisms

The capacity of deep subsurface microorganisms to convert CO_2_ into liquid products is also determined by substrate availability and competitive interactions within the community. In mixed cultures, different microorganisms compete for CO_2_ and reducing equivalents, making community regulation essential for improving selectivity toward target products. For instance, methanogenic archaea and homoacetogenic bacteria often compete for the same H_2_-CO_2_ substrates,^14^ and from a Gibbs free energy perspective, methanogenesis is typically favored. By shifting environmental conditions away from those optimal for methanogens, or by adding selective inhibitors (such as 2-bromoethanesulfonate), methanogenic activity can be suppressed—allowing homoacetogens to become dominant and thus enhancing the yield of liquid products like acetate.^14^^,^^206^

**Figure 19:**
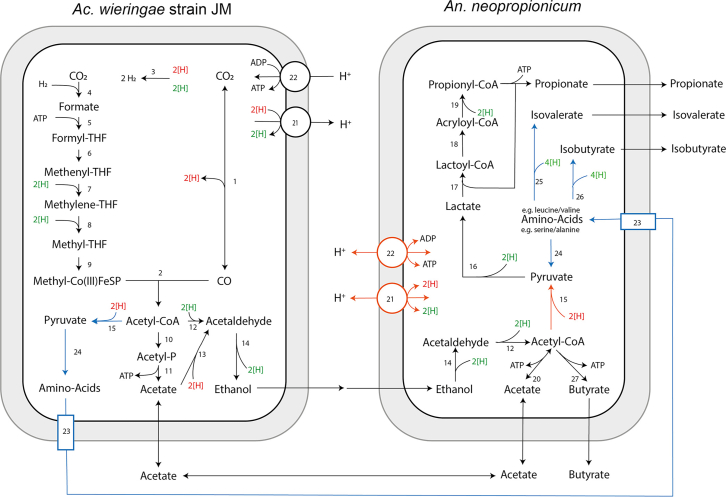
Schematic representation of the metabolic pathway for conversion of CO/CO_2_ to propionate via acetate/ethanol intermediates (reprinted from Moreira et al.^224^ licensed under CC BY 4.0.)

On the other hand, constructing functionally complementary coculture systems can harness the synergistic effects of different functional strains, leading to more efficient metabolic handoffs and a broader spectrum of liquid products derived from CO_2_. For example, Moreira et al.^224^ developed a synthetic coculture of *Acetobacterium* (homoacetogen) and a propionate-producing bacterium. In this system, *Acetobacterium* first converts CO/CO_2_ to acetate and ethanol, after which the propionate producer utilizes ethanol as the electron donor and CO_2_ or acetate as a carbon source to synthesize propionate via the acrylate pathway. Proteomic analyses revealed that the two strains may exchange metabolites such as amino acids, promoting the formation of branched-chain organic acids (Figure 19). Similarly, to obtain longer-chain liquid products, Wu et al.^232^ applied a two-stage process in which anaerobic homoacetogens first reduce CO_2_ to precursors such as acetate and ethanol, followed by chain-elongating bacteria (e.g., *Clostridium kluyveri*) converting these into medium-chain fatty acids (e.g., caproate, octanoate, etc.), significantly enhancing both system stability and overall yield.

#### Advances in process research and product conversion potential

At present, microbial bioliquefaction of CO_2_ remains largely at the laboratory or pilot scale, with product yields and conversion efficiencies still falling short of requirements for large-scale practical application. Nevertheless, several demonstration projects using industrial off-gases as a carbon source have preliminarily verified the feasibility of converting CO_2_ into liquid fuels via microbial processes. For example, companies such as LanzaTech have successfully operated scalable pilot facilities that employ anaerobic clostridia fermentation technology to convert CO/CO_2_-rich steel mill waste gases into ethanol.^223^ Additionally, syngas fermentation technologies have enabled the conversion of industrial off-gases into liquid products such as acetate. As an important platform chemical for biorefining, acetate holds significant promise for industrial-scale applications.^233^

Emerging microbial electrosynthesis (MES) technologies have further improved the efficiency of CO_2_-to-ethanol conversion. MES uses cathodic biofilms to catalyze the electrochemical reduction of CO_2_, with daily ethanol production rates reaching up to 10.95 g per square meter of electrode surface area.^234^ Through metabolic engineering and process optimization, the range of microbial CO_2_ conversion products is expanding from simple C_2_ compounds (e.g., ethanol, acetate) to higher-value C_3_ and longer-chain organics, including 2,3-butanediol,^235^ propionate,^224^ and medium-chain fatty acids (C_6_–C_12_).^232^

From the perspective of subsurface sequestration, *in situ* microbial bioliquefaction of CO_2_ has received preliminary support from laboratory and field studies. For example, carbon and hydrogen isotope analyses in the deep formations of the Powder river basin in the United States have indicated that a portion of acetate salts are produced *in situ* by autotrophic acetogenic bacteria.^236^ Ohtomo et al.,^207^ using temperature and pressure simulation experiments, further demonstrated that CO_2_ injection into geological formations preferentially stimulates bacteria to produce acetate, exhibiting distinct homoacetogenic metabolic characteristics. Additionally, the injection of CO_2_ into storage formations may promote the leaching of ferrous iron from minerals such as magnetite, which can further catalyze the reduction of CO_2_ to acetate.^237^

#### Engineering challenges and future perspectives

Despite the promising potential of CO_2_ bioliquefaction, several key technical challenges must be addressed for its practical application in GCS. One major issue is the feedback inhibition caused by the accumulation of liquid products—primarily acetate—in the reservoir over long timescales, which can suppress the activity of acetogenic bacteria.^228^ In addition, the generation of byproduct gases requires special attention; for example, while methane production offers resource utilization value, it may also increase the risk of gas leakage from the storage formation, and the production of hydrogen sulfide through sulfate reduction poses significant environmental hazards.^110^

The long-term persistence of microbial reactions and the sustained supply of nutrients are also critical concerns. GCS operates over extended time frames, and microbial activity depends on a continuous supply of electron donors and nutrients.^238^ Changes in reservoir conditions, such as temperature and CO_2_ saturation, may lead to reduced microbial viability and activity over time.^239^ Additionally, the proliferation of microbial biomass and accumulation of metabolic byproducts may cause pore clogging, further affecting storage efficiency.^110^

Future research should focus on elucidating the mechanisms of deep subsurface CO_2_ bioliquefaction, evaluating the kinetic responses of different reservoirs, and developing robust strategies for long-term conversion. Notably, the integration of MES systems powered by renewable electricity—to directly capture and convert industrial flue gas CO_2_—holds promise for efficient coupling of carbon capture and utilization in engineered settings.

### CO_2_ biominineralization

CO_2_ biominineralization refers to the process by which microbial metabolic activity induces the precipitation of carbonate minerals, thereby immobilizing CO_2_ in mineral form within GCS environments. The fundamental principle of MICP is that certain microorganisms produce alkaline substances or promote CO_2_ hydrolysis, which increases local pH and generates carbonate ions; in the presence of Ca^2+^, these ions precipitate as stable minerals such as calcium carbonate.^106^^,^^240^

Currently, the most widely applied CO_2_ biominineralization pathway is urease-driven urea hydrolysis, primarily mediated by ureolytic bacteria (e.g., *Sporosarcina pasteurii*). These microorganisms secrete urease, catalyzing the hydrolysis of urea into ammonia and bicarbonate. The produced NH_3_ is rapidly hydrated to NH_4_^+^ and OH^−^, resulting in a rise in environmental pH and providing CO_3_^2−^ for CaCO_3_ precipitation when Ca^2+^ is present.^15^ Other commonly reported pathways include uric acid hydrolysis, sulfate reduction, denitrification, and carbonic anhydrase-catalyzed processes; all can induce CaCO_3_ precipitation through different mechanisms, thus enabling long-term and secure mineral trapping of CO_2_ in geological formations.^89^^,^^96^^,^^208^

#### Influence of formation conditions on microbial mineralization reactions

MICP in geological CO_2_ sequestration is strongly influenced by reservoir environmental conditions, with pH being a critical factor. Injected CO_2_ dissolves in groundwater to form carbonic acid, which significantly lowers the system pH and restricts the activity of most microorganisms. However, certain microorganisms, such as ureolytic bacteria, can locally raise the pH to alkaline conditions via urea hydrolysis, thereby enabling significant calcium carbonate precipitation.^15^ When the pressure exceeds the supercritical CO_2_ threshold (>75 bar), the capacity of microorganisms to modulate pH is greatly reduced, resulting in markedly decreased mineralization efficiency.^106^

In addition to pH, calcium ion concentration has a major impact on mineralization. Natural reservoir Ca^2+^ concentrations are often insufficient to support large-scale precipitation, so external calcium sources are typically required. While moderate increases in Ca^2+^ concentration can effectively enhance mineralization rate and precipitate volume, excessively high concentrations may lead to clogging near the injection well, reducing the spatial extent of the reaction.^241^

Temperature is another key factor affecting both microbial activity and mineralization rate. The optimal growth temperature for most ureolytic bacteria is 20°C–40 °C, whereas temperatures above 60 °C tend to inactivate conventional strains.^72^^,^^242^ However, some thermophilic strains, such as *Bacillus haynesii*, remain active above 50 °C and are thus suitable for high-temperature deep reservoir environments.^243^

Reservoir permeability and pore structure also significantly affect the spatial distribution of MICP.^244^ High initial permeability facilitates bacterial dispersion, but as CaCO_3_ precipitates, permeability decreases, often resulting in clogging near the wellbore. Groundwater salinity, ionic strength, and redox conditions further influence mineralization pathways and efficiency.^245^^,^^246^ Moderate salinity can enhance microbial metabolism and accelerate mineralization. Redox conditions determine dominant metabolic pathways: aerobic conditions favor urea hydrolysis, while anaerobic conditions enable sulfate reduction and denitrification. Byproducts such as hydrogen sulfide and ammonia, however, may cause pollution or corrosion.

#### Sequestration enhancement and leakage control mechanisms

MICP not only enables the mineral sequestration of CO_2_ but also shows great potential for improving reservoir storage performance and safety.^208^ Carbonate minerals (e.g., calcium carbonate) produced during MICP can effectively fill reservoir pores and fractures, significantly reducing porosity and permeability, and forming a dense mineral matrix that impedes CO_2_ migration along leakage pathways.^247^ Experimental studies have shown that microbial mineralization treatment can reduce reservoir permeability by 2-4 orders of magnitude, greatly enhancing the reservoir’s capacity to retain fluids and thus providing an additional physical barrier for geological sequestration.^248^^,^^249^ As the reaction time progresses from 6 to 54 h, microbially induced CaCO_3_ precipitates evolve from nanoscale amorphous calcium carbonate to interconnected microscale calcite clusters, continuously filling and clogging pore throats. The morphological evolution corresponds closely with the sharp decrease in permeability.^250^

In addition, microbial mineralization technology is especially advantageous for repairing microfractures, faults, and wellbore interfaces in the reservoir and caprock.^251^ Traditional plugging methods, such as cement slurry, have high viscosity and poor penetration into micro-pores or fine fractures; by contrast, microbially induced mineral precipitation utilizes low-viscosity nutrient and bacterial solutions that can infiltrate fine cracks and form mineral deposits deep within, thus achieving effective leakage control.^252^^,^^253^ Field studies by Phillips et al.^252^ have shown that after multiple rounds of MICP treatment, fracture permeability within the reservoir was reduced by about 75%, significantly lowering the risk of CO_2_ leakage. Furthermore, mineralization enhances the mechanical strength and integrity of the reservoir-caprock system, reducing the likelihood of failure due to subsequent geological disturbances and thus ensuring long-term sequestration stability.^254^

From a long-term perspective, CO_2_ sequestered via mineralization remains stable for hundreds to thousands of years, even in the event of pressure or temperature changes in the geological environment. This is in stark contrast to free-phase or dissolved CO_2_, which is much more susceptible to escape under changing conditions, thereby greatly improving the long-term security of geological carbon storage.^255^

#### Process intensification of MICP

Given the relatively low efficiency and slow reaction rate of CO_2_ MICP, recent research has focused on a suite of intensification strategies to enhance the efficiency and spatial uniformity of carbonate precipitation. Among the most direct approaches, optimizing injection protocols and controlling reaction conditions have proven effective. Extending the residence time for nutrient and microbial injection or increasing the injection flow rate can improve the uniform distribution of microbes and minimize premature carbonate accumulation near the injection site, thereby promoting deeper precipitation within the formation.^16^ Adjusting the initial pH of injection fluids can also help control the timing and locus of precipitation, fostering more homogeneous mineralization throughout the reservoir.^256^ The addition of exogenous nucleating agents, such as montmorillonite or quartz sand particles, has been shown to increase crystal nuclei density and promote more uniform and rapid CaCO_3_ deposition.^257^

Enzyme-mediated intensification represents another promising avenue: genetic engineering to co-express urease and carbonic anhydrase, or the use of mixed functional consortia, can significantly boost overall mineralization efficiency.^16^^,^^74^ Electrochemical enhancement is also emerging as a powerful method; weak electric fields (∼0.5 V/cm) have been shown to stimulate the metabolic activity of *Sporosarcina pasteurii* and increase the yield and regularity of CaCO_3_ crystals.^257^^,^^258^

Coupled process strategies are gaining traction as well—for example, integrating photoautotrophic carbon fixation with heterotrophic precipitation in tandem with electrodialysis pH regulation, thus synchronously intensifying both carbon capture and mineralization.^257^ Temperature-regulated MICP processes have also been demonstrated to precisely control the locus of precipitation and improve the spatial uniformity and stability of mineralization.^259^ Incorporating adsorptive materials such as zeolite during MICP can help capture ammonium by-products, reduce environmental risk, and further enhance the mechanical performance of the precipitated minerals.^260^

#### Engineering challenges and future perspectives

Despite the promising potential of MICP for geological carbon sequestration, scaling up from laboratory to field-scale applications faces several significant engineering challenges. The most prominent obstacles are limited process controllability and non-uniform mineralization, which often leads to precipitation concentrated near injection wells, resulting in reduced overall storage efficiency or even local clogging.^261^ Additionally, the inherently slow mineralization rate is a key barrier for large-scale CO_2_ treatment, as microbial growth, metabolism, and environmental adaptation limit the overall process speed compared to purely chemical pathways.^262^ The harsh conditions of deep geological environments—high pressure, elevated temperature, and high salinity—also pose severe challenges to microbial survival and activity, with most existing strains unable to remain active over long periods.^208^ Moreover, environmental impacts arising from process by-products, such as ammonium ions generated via urea hydrolysis, raise concerns about potential groundwater contamination.^263^

**Figure 20:**
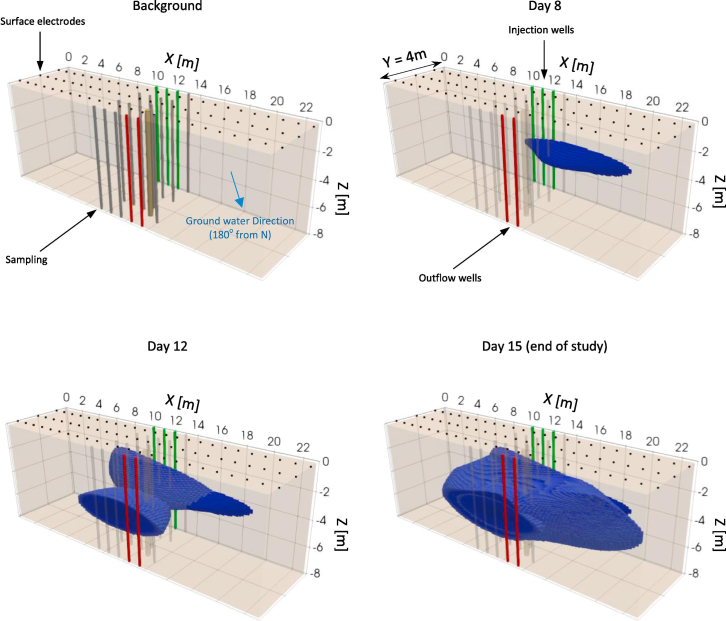
Conceptual model of MICP progress based on induced polarization imaging (reprinted from Saneiyan et al.^266^ Copyright 2019, Elsevier.)

Looking forward, microbially mediated mineralization may offer potential value in selected GCS settings, but its practical relevance remains strongly site-dependent and will require advances in process optimization, long-term stability assessment, and comprehensive environmental risk evaluation. For instance, genetically engineered strains have been proposed as a potential route to integrate multiple metabolic functions and improve mineralization efficiency while reducing by-product generation^242^^,^^264^; however, their applicability in deep subsurface GCS environments remains highly uncertain and requires careful biosafety, stability, and deployment assessment. Enzyme engineering may also provide alternative catalytic strategies (e.g., for CO_2_ hydration),^265^ but their persistence, transport behavior, and effectiveness under reservoir-relevant temperature, pressure, and salinity conditions still require validation. On the engineering side, smart control systems and real-time monitoring technologies could support adaptive adjustment of injection parameters, potentially improving alignment between precipitation behavior and engineering design targets,^266^^,^^267^ provided that field-scale monitoring reliability and controllability can be demonstrated. As illustrated in Figure 20, Saneiyan et al.^266^ constructed an induced polarization imaging-based conceptual model to track MICP progress and the spatial evolution of calcite precipitation over 8–15 days. This type of approach provides a useful methodological reference for data-assisted process monitoring and control, although its transferability to deep GCS field conditions requires further validation.

## Microbial considerations for GCS implementation

### Site selection

Site selection for GCS may benefit from considering subsurface microbial communities and their potential impacts under site-specific reservoir conditions. Depending on reservoir conditions, microbial activities may be relevant to specific transformation or risk pathways, whereas in many deep storage settings their effects may be limited; when active, unfavorable processes can compromise storage performance or safety. For example, a field study at the Olla oil field (Louisiana, USA)—a CO_2_-EOR, hydrocarbon-bearing setting with conditions favorable to microbial activity—reported that 13%–19% of injected CO_2_ was converted to methane by indigenous microbes, while most of the remainder dissolved in the aquifer.^10^ As noted by the original authors, such observations are most relevant to reservoirs that fall within an “environmental window” conducive to microbial methanogenesis (e.g., relatively lower temperature/salinity and sufficient substrate availability), and therefore should be interpreted as a conditional site-screening consideration rather than a generally representative pathway for dedicated deep saline GCS.^10^ Similarly, where sulfate-reducing microbial activity is sustained, corrosive H_2_S generation and/or bioclogging may reduce injection capacity and effective porosity.^90^^,^^110^ Bonto et al.^110^ summarized such microbially mediated risks and highlighted that reservoir mineralogy, groundwater chemistry, and injection conditions all influence microbial activity. Accordingly, site selection should incorporate microbial risk screening where relevant and prioritize reservoir/injection conditions that minimize the likelihood of adverse microbial processes.

**Figure 21:**
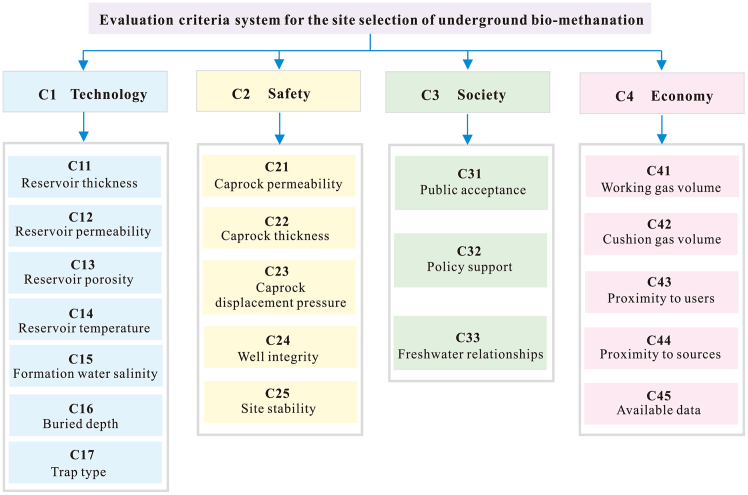
Evaluation framework for site selection in underground biological methanation (reprinted from Wu et al.^269^ Copyright 2024, Elsevier.)

Traditionally, GCS site selection has focused on geological and engineering criteria such as storage capacity, seal integrity, injectivity, and trapping efficiency. For instance, Rasool et al.^268^ comprehensively ranked different reservoir types based on seven factors (including safety, storage capacity, injection rates, efficiency, and residual trapping), finding that deep saline aquifers and basalt formations generally scored highest. However, such studies rarely consider microbial factors, despite increasing evidence that CO_2_ injection can alter subsurface microbial ecology in some settings, with potential implications for geochemical reactions and long-term storage evolution.^8^^,^^200^ Therefore, incorporating selected microbial indicators (e.g., baseline community profiles) together with key reservoir constraints (e.g., temperature and salinity) into site screening may help identify and mitigate microbial risks where such processes are likely to be relevant.^8^^,^^110^ Wu et al.^269^ further proposed a comprehensive site assessment framework for underground biological methanation of CO_2_ and H_2_, integrating technical, safety, societal, and economic factors (Figure 21).

### Microbial engineering and process control

From a forward-looking and site-specific perspective, microbial control strategies have been proposed as potential approaches for influencing subsurface microbial metabolism in ways that may affect CO_2_ transformation or storage-related processes; however, their practical applicability in GCS remains limited by reservoir conditions, operational controllability, and techno-economic constraints.

Nutrient amendment is one proposed approach for stimulating *in situ* microbial growth in bio-reactive subsurface systems.^270^ For example, in biomethanation trials involving high-sulfate aquifers, the background concentrations of NH_4_^+^ and PO_4_^3−^ were only a few μM, limiting microbial proliferation,^271^ highlighting the necessity of co-injecting nutrient solutions containing N and P in real applications. In carbon-rich deep formations, hydrogenotrophic methanogenesis is considered a primary CO_2_ fixation pathway.^272^ Co-injection of CO_2_ with hydrogen or fermentable organics may enhance microbial methanation under favorable conditions, but it also introduces additional operational, safety, and economic considerations. However, such stimulation strategies are highly context-dependent and may be more applicable to selected hydrocarbon-bearing or biomethanation-oriented scenarios than to typical deep saline GCS operations.

Operational parameters and reservoir environment are equally critical for controlling biological processes. Increasing injection pressure can substantially improve CO_2_ solubility in aquifers, while reducing salinity accelerates CO_2_ dissolution into the aqueous phase.^20^ Both simulation and field studies (e.g., Shchipanov et al.^273^) demonstrate that cyclic or staged injection, as opposed to continuous injection, induces a “CO_2_ expansion-squeeze” effect that helps manage reservoir pressure and increase storage capacity. Other process adjustments, such as changing injection fluid phase (e.g., CO_2_-water two-phase flow), regulating injection temperature, or adding pH buffers, may also influence microbial habitat conditions and geochemical responses; their effects should be evaluated with respect to both storage performance and microbial risk control.

Community engineering (e.g., selection or inoculation of functional consortia) has been discussed as a possible way to steer metabolic pathways in subsurface bio-reactive systems, but its feasibility and controllability in deep GCS reservoirs remain uncertain. For example, co-cultures of facultative fermenters and methanogens can achieve multi-step conversion of organic substrates and continuous utilization of CO_2_. In simulated high-sulfate conditions (∼150 μM), Ranchou-Peyruse et al.^271^ observed that about 80% of injected H_2_ was consumed by methanogens, with only minor H_2_ assimilation by other bacteria, confirming the dominant role of methanogens in H_2_ utilization. In practice, lowering sulfate concentration or supplementing essential metals (e.g., Ni^2+^) can inhibit competing sulfate reduction or enhance methanogenic activity,^271^ and targeted inoculation of high-efficiency methanogenic strains can further improve methane yields.

Overall, these approaches should be viewed as conditional and largely exploratory options, requiring site-specific validation and careful assessment of reservoir-condition constraints, operational controllability, storage integrity risks, and techno-economic feasibility before any field-scale application is considered.

### Field monitoring and risk control

Beyond listing potential negative impacts (e.g., clogging and corrosion), microbial risks in GCS should be evaluated using a simple but systematic workflow that links monitoring to decision-making.^61^^,^^274^ In practice, this workflow includes: (1) identifying the dominant microbial-induced risk pathways (e.g., injectivity impairment, souring/corrosion, or undesired gas transformation), (2) screening reservoir and operational susceptibility based on site conditions (temperature, salinity, pressure, pH, nutrient/electron donor availability, and injection strategy), (3) defining quantifiable indicators and baseline values (e.g., injectivity decline, pressure increase, H_2_S concentration, gas composition changes, and microbial community shifts), and (4) implementing adaptive management measures when anomalies are detected. This process helps convert microbial monitoring from a descriptive activity into a risk-informed management tool for storage safety and performance.

Representative observations further illustrate how microbial-related risks can be quantified and managed in practice. For example, contamination during drilling and sampling can bias microbiological interpretation if not controlled; therefore, tracer-based contamination assessment and threshold-based sample cleaning/selection are essential for reliable risk diagnosis.^190^ In addition, field evidence of microbial gas transformation (e.g., methanogenesis under favorable conditions) suggests that gas composition changes can be tracked as quantifiable indicators and incorporated into site screening and monitoring strategies.^10^ These examples indicate that microbial risks should be managed through integrated monitoring, baseline comparison, and site-specific operational response rather than by microbial measurements alone.

Compared with traditional geophysical and geochemical monitoring, biological monitoring can provide complementary early-warning signals for GCS. Park et al.,^275^ for instance, combined CO_2_ flux/concentration, soil pH, and 16S rRNA-based microbial community analyses at a deep CO_2_ leakage site in Korea, and found that elevated CO_2_ flux was associated with soil acidification and microbial community shifts, suggesting that coupled biological-geochemical indicators may support leakage detection. Some studies also propose groundwater microbial community monitoring in observation wells to help trace deep CO_2_ migration.^276^ Emerging approaches, including DNA-sequencing-based microbial tracing and biosensors, may further improve monitoring sensitivity and spatial/temporal resolution for detecting leakage-related environmental changes.^277^^,^^278^

Taken together, current evidence suggests that microbial monitoring and control in GCS should primarily be framed as a site-screening and risk-management issue, rather than a universally applicable performance-enhancement strategy.

## Conclusions and future perspectives

### Conclusions

GCS is recognized as a crucial approach for mitigating climate change, with subsurface microbial processes exerting multifaceted impacts on the fate and long-term security of CO_2_ storage. Microorganisms participate in the deep carbon cycle primarily through three interconnected mechanisms: biomethanation, which enables the energetic recycling of CO_2_; bioliquefaction, which facilitates the transformation of *in situ* organic carbon; and biomineralization, which provides the potential for permanent, stable sequestration. While these processes are complementary and often mutually reinforcing, variations in reservoir conditions can also lead to negative effects, such as pore clogging or the production of corrosive gases.

To assess microbial roles in GCS, this review has synthesized findings from laboratory experiments, numerical simulations, and field observations. Laboratory studies elucidate the coupling mechanisms between microbial metabolism and CO_2_ geochemistry; numerical models extend these micro-scale insights to the reservoir scale for long-term behavioral predictions; and field studies validate the occurrence and scale effects of microbial processes in real-world reservoirs. This integrated “experiment-simulation-field” framework reveals the feedback chain of “reservoir environment-microbial response-carbon fixation”: reservoir conditions dictate community structure and metabolic activity, while microbial processes reshape pore architecture, flow pathways, and chemical environments, ultimately affecting the stability of CO_2_ storage.

Consequently, the implementation of GCS must fully account for microbial factors. Site selection should incorporate microbial ecological risk assessments to ensure favorable conditions for beneficial functions. Engineering strategies should emphasize nutrient provision, co-injection of electron donors, and community optimization to improve storage efficiency. In addition, microbial community-based biosurveillance offers sensitive detection of leakage signals, enhancing the long-term safety of storage operations.

### Future research directions

Although the prospects for microbial applications in GCS are increasingly recognized, current research remains at an early stage, especially in terms of field validation, parameterization, and engineering integration. To provide clearer guidance, future research priorities can be organized into short-, mid-, and long-term horizons according to current evidence gaps, technology readiness, and practical needs.

Short-term priorities (<5 years) should focus on strengthening the reliability and comparability of evidence across laboratory, modeling, and field studies. First, laboratory research should better mimic reservoir-relevant conditions by constructing systems capable of stable operation under high temperature, high pressure, high salinity, and high CO_2_ concentration, while also considering substrate limitation and impurity effects (e.g., SOx, NOx, O_2_, etc.) on microbial activity.^67^ Second, more field-relevant experimental designs are needed to investigate synergistic mechanisms among metabolic pathways in complex geochemical environments and to assess the effects of different electron donors on methanation, liquefaction, and mineralization. Third, standardized protocols for deep subsurface sampling, contamination control, and sample preservation should be further developed and adopted to improve microbiological data reliability in GCS-related studies. In parallel, practical microbial risk indicators (e.g., injectivity/pressure changes, gas composition changes, and microbial community shifts) and baseline datasets for representative reservoir types should be progressively established. Advanced tools such as microfluidics can also be introduced to simulate deep pore structures at the microscale and enable real-time observation of microbial metabolism and mineral-fluid interface reactions.^279^

Mid-term priorities (5–10 years) should emphasize cross-scale validation and predictive capability. Numerical modeling remains constrained by high parameter uncertainty and the difficulty of quantifying microbial kinetic processes; therefore, more comprehensive bio-reactive transport coupling models are needed to improve prediction from pore to reservoir scale. A key priority is to strengthen parameter calibration and model validation through tighter integration of experimental data, field observations, and multi-scale monitoring results. Comparative analyses across reservoir types and demonstration sites should be expanded to identify where microbial effects are negligible, moderate, or operationally significant under different geological and operational conditions. In addition, hybrid approaches that combine machine learning with conventional numerical modeling may help reduce computational loads and improve the efficiency of scenario analysis and near-real-time prediction.^280^ During this stage, risk-based monitoring frameworks that integrate microbial, geophysical, and geochemical indicators should also be further refined for decision support.

Long-term priorities (>10 years) should focus on conditional engineering application and full-system integration, where supported by robust field evidence and techno-economic assessment. This includes evaluating the site-specific feasibility of intentionally leveraging microbial processes (e.g., controlled biomethanation, bioliquefaction enhancement, or biomineralization-assisted trapping) in suitable reservoir settings, while fully accounting for risks such as clogging, souring/corrosion, and gas composition changes. Long-term efforts should also integrate microbial ecological considerations into site screening, operational management, and adaptive risk control strategies for GCS projects.^8^^,^^269^ At the system level, establishing a comprehensive subsurface microbial database—integrating community composition, metabolic functions, reservoir conditions, and geological features—together with stronger interdisciplinary collaboration among microbiology, geochemistry, rock physics, and numerical modeling, will be essential for building reliable predictive frameworks and best-practice guidance for microbially informed GCS engineering.

In summary, future progress in this field will depend on coordinated advances in field-relevant experimentation, predictive modeling, long-term monitoring, and interdisciplinary data integration. Such efforts are necessary to clarify when microbial processes in GCS are beneficial, negligible, or adverse, and to support safer and more controllable storage system design and management.

## Acknowledgments

This work was supported by the Henan Centre for Outstanding Overseas Scientists in Green Low-Carbon Technology and Energy Transition (grant no. GZS2024001), European Union’s “Horizon Europe programme”- LOC3G (grant no. 101129729) and the Soft Science Major Project of Henan Province (grant no. 242400411004).

## Author contributions

L.H.: writing – review and editing, writing – original draft, formal analysis, and conceptualization; Z.H.: writing – review and editing, supervision, project administration, and funding acquisition; J.L.: funding acquisition and supervision. T.S.: writing – review and editing, methodology, and visualization; Q.W.: investigation and formal analysis; Y.G.: formal analysis and data curation; L.W.: writing – review and editing, visualization, and formal analysis.

## Declaration of interests

The authors declare no competing interests.
